# Efficacy and safety of the herbal formula *Naesohwajung-tang* for functional dyspepsia: a randomized, double-blind, placebo-controlled, multi-center trial

**DOI:** 10.3389/fphar.2023.1157535

**Published:** 2023-05-12

**Authors:** Na-Yeon Ha, Seok-Jae Ko, Jae-Woo Park, Jinsung Kim

**Affiliations:** ^1^ Division of Digestive Diseases, Department of Internal Korean Medicine, Kyung Hee University Medical Center, Seoul, Republic of Korea; ^2^ Department of Gastroenterology, Kyung Hee University College of Korean Medicine, Kyung Hee University Hospital at Gangdong, Seoul, Republic of Korea; ^3^ Department of Gastroenterology, Kyung Hee University College of Korean Medicine, Kyung Hee University Medical Center, Seoul, Republic of Korea

**Keywords:** functional dyspepsia, randomized controlled trial, *Naesohwajung-tang*, electrogastrography, pattern identification, herbal medicine

## Abstract

**Background:** Current treatment and management options for functional dyspepsia (FD) often fail to alleviate symptoms. *Naesohwajung-tang* (NHT) is a herbal formula frequently used to treat functional dyspepsia in traditional Korean medicine. However, few animal and case reports on the use of *Naesohwajung-tang* for functional dyspepsia treatment exist, and the clinical evidence remains deficient.

**Objectives:** This study aimed to evaluate the efficacy of *Naesohwajung-tang* in patients with functional dyspepsia.

**Methods:** We enrolled 116 patients with functional dyspepsia at two study sites in this 4 weeks, randomized, double-blind, placebo-controlled trial and randomly assigned them to either the *Naesohwajung-tang* or placebo group. To evaluate the efficacy of *Naesohwajung-tang*, the primary endpoint was a score on the total dyspepsia symptom (TDS) scale after treatment. The overall treatment effect (OTE), single dyspepsia symptom (SDS) scale, food retention questionnaire (FRQ), *Damum* questionnaire (DQ), functional dyspepsia-related quality of life (FD-QoL) questionnaire, and gastric myoelectrical activity measured using electrogastrography were evaluated as secondary outcomes. Laboratory tests were performed to confirm the safety of the intervention.

**Results:** The 4 weeks administration of *Naesohwajung-tang* granules demonstrated a significantly higher reduction in the total dyspepsia symptom (*p* < 0.05) and a higher degree of improvement in the total dyspepsia symptom (*p* < 0.01) than the placebo group. Patients who underwent *Naesohwajung-tang* had a significantly higher overall treatment effect and a greater increase in the degree of improvement in scores such as epigastric burning, postprandial fullness, early satiation, functional dyspepsia-related quality of life, and *Damum* questionnaire (*p* < 0.05). Additionally, the *Naesohwajung-tang* group showed a greater effect in preventing a decrease in the percentage of normal gastric slow waves after meals than the placebo group. As a result of subgroup analyses using the degree of improvement in total dyspepsia symptom, *Naesohwajung-tang* was found to be more effective than placebo in female, younger patients (<65 years), with a high body-mass index (≥22), overlap type, food retention type, and Dampness and heat in the spleen and stomach systems pattern. There was no significant difference in the incidence of adverse events between the two groups.

**Conclusion:** This is the first randomized clinical trial to verify that *Naesohwajung-tang* leads on symptom relief in patients with functional dyspepsia.

**Clinical Trial Registration:** https://cris.nih.go.kr/cris/search/detailSearch.do/17613, identifier KCT0003405

## 1 Introduction

Patients with functional dyspepsia (FD) complain of at least one of the following symptoms for more than 6 months prior to diagnosis: postprandial fullness, early satiation, epigastric pain, or epigastric burning. According to the Rome IV criteria, the international standard diagnostic criteria for functional gastrointestinal disorders (FGIDs), FD is diagnosed when the patient’s clinical symptoms meet the criteria while excluding organic causes of dyspepsia, including peptic ulcer disease, gastroesophageal malignancy, and medications (Stanghellini et al., 2016). The worldwide prevalence of FD is reported to be 11%–30% (Mahadeva and Goh, 2006). Factors such as dietary habits, sociocultural differences, psychological issues, and gastrointestinal (GI) infections affect dyspeptic symptoms, resulting in differences in the distribution of prevalence (Ghoshal et al., 2011). Although the key pathogenesis of FD is not clear, various mechanisms are known to act in combination, including gastric dysmotility, such as gastric emptying and accommodation (Park et al., 2017), visceral hypersensitivity (Vanheel et al., 2017), *Helicobacter pylori* infection (Moayyedi et al., 2005), and psychosocial dysfunction, such as anxiety and depression (Mak et al., 2012). FD can be classified into three subtypes based on the predominant symptoms: 1) postprandial distress syndrome (PDS), characterized by aggravating postprandial fullness and/or early satiation symptoms; 2) epigastric pain syndrome (EPS), characterized by aggravating epigastric pain or burning symptoms; and 3) overlap type, characterized by the coexistence of PDS and EPS (Carbone et al., 2015). The PDS type is more common in Asia than in North America or Europe (Mahadeva and Ford, 2016). FD extensively affects the health-related quality of life in terms of physical, psychological, and social aspects (Aro et al., 2011). Moreover, it causes direct and indirect costs owing to its long-lasting and recurrent symptoms, which reduce productivity.

Although various treatments including *H. pylori* eradication therapy (Mazzoleni et al., 2011), proton pump inhibitors (PPIs) (Pinto-Sanchez et al., 2017), H_2_ receptor antagonists (H_2_RA) (Moayyedi et al., 2006), antidepressants (Jackson et al., 2000), and prokinetic agents (Pittayanon et al., 2019) have been devised to manage symptoms in patients with FD, current treatments for FD are limited because the underlying causes of the condition are not fully understood (Masuy et al., 2019). FD often shows a chronic course, and its symptoms vary widely over a long period of time (Talley and Ford, 2015). Herbal medicine, a representative therapy in the realm of complementary and alternative medicine, comprises a diverse array of active ingredients. As a result, it can simultaneously target multiple pathophysiological mechanisms, such as GI motility, secretory function, cellular protection, and psychoactive properties (Gwee et al., 2021). As these advantages have been highlighted, the potential of herbal medicine as a new treatment for FD, which is complex and involves various etiologies, has been recognized. Specifically, 25% of patients with FD in the United States tried alternative therapies, and their expenditures reached $111 (range $0–3,741) (Lacy et al., 2013).

*Naesohwajung-tang* (NHT) is one of the most frequently used herbal medicine in traditional Korean medicine (TKM) for the treatment of FD (Table 1). NHT, composed of 19 single botanical drugs, is a combination of two well-known herbal formulas: *Naeso-san* (NSS) and *Daehwajung-eum* (DHJ). In a previous study, 4 weeks administration of NHT in children with FD experiencing abdominal pain improved abnormal electrogastrography (EGG) parameters including the rate of normogastric and dominant power (DP) in the postprandial phase (Kim et al., 2002). Based on these results, NHT was expected to ameliorate dysmotility-related FD symptoms by regulating gastric motility. However, no randomized clinical trials (RCTs) of high methodological quality have been conducted to provide objective evidence for NHT as a treatment option for patients with FD. This is the first randomized placebo-controlled study to evaluate the safety and clinical efficacy of NHT in adult patients with FD.

**TABLE 1:** Composition and dosage of NHT.

No.	Scientific name (family)	Chinese name	Dosage per serving (g)
1^a^ ^,^ ^b^	*Crataegus pinnatifida* Bunge (family *Rosaceae*)	Shanzha	2.50
2^b^	*Hordeum vulgare* L. (family *Poaceae*)	Chao-Maiya	2.50
3^b^	*Magnolia officinalis* Rehder & E.H.Wilson (family *Magnoliaceae*)	Houpo	1.88
4^a^ ^,^ ^b^	*Citrus reticulata* Blanco (family *Rutaceae*)	Chenpi	1.88
5^b^	*Alisma plantago-aquatica* subsp. *orientale* (Sam.) Sam. (family *Alismataceae*)	Zexie	1.88
6	*Atractylodes lancea* (Thunb.) DC. (family *Compositae*)	Cangzhu	1.25
7^a^	*Cyperus rotundus* L. (family *Cyperaceae*)	Xiangfu	1.25
8^a^ ^,^ ^b^	*Citrus trifoliata* L. (family *Rutaceae*)	Zhishi	1.25
9^a^	*Pinellia ternata* (Thunb.) Makino (family *Araceae*)	Banxia	1.25
10^a^	*Poria cocos* (Schw.) Wolf. (family *Polyporaceae*)	Fuling	1.25
11^a^	—	Chao-Shenqu	1.25
12^a^ ^,^ ^b^	*Wurfbainia villosa* (Lour.) Škorničk. & A.D.Poulsen (family *Zingiberaceae*)	Sharen	1.25
13^a^	*Sparganium stoloniferum* (Buch.-Ham. ex Graebn.) Buch.-Ham. ex Juz. (family *Typhaceae*)	Sanleng	1.25
14^a^	*Curcuma phaeocaulis* Valeton (family *Zingiberaceae*)	Ezhu	1.25
15^a^	*Zingiber officinale* Roscoe (family *Zingiberaceae*)	Ganjiang	1.25
16	*Z. officinale* Roscoe (family *Zingiberaceae*)	Shengjiang	1.25
17	*Agastache rugosa* (Fisch. & C.A.Mey.) Kuntze (family *Lamiaceae*)	Huoxiang	1.00
18	*Aucklandia lappa* DC. (family *Compositae*)	Muxiang	0.67
19	*Glycyrrhiza uralensis* Fisch. (family *Leguminosae*)	Gancao	0.67

^a^ 11 botanical drugs included in *Naeso-san*.

^b^ 7 botanical drugs included in *Daehwajung-eum*.

NHT, *Naesohwajung-tang*.

## 2 Materials and methods

### 2.1 Study design

This was a prospective, multi-center, randomized, double-blind, parallel-group, placebo-controlled trial. This clinical trial with a 4 weeks NHT administration aimed to determine whether NHT improves clinical symptoms in patients with FD compared to placebo. The protocol for our recent study described the study information in detail, including sample size calculation, forbidden drugs, criteria for withdrawal, and protocol for EGG recording (Ha et al., 2020), strictly following the Consolidated Standards of Reporting Trials (CONSORT) guidelines (Schulz et al., 2010).

### 2.2 Participants

A total of 116 participants with FD were recruited from two study sites, Kyung Hee University Medical Center and Kyung Hee University Hospital at Gangdong, Seoul, Republic of Korea, from 2019 to 2020. The protocol was approved by the Institutional Review Board of each study center (approval number KOMCIRB-2017-08-030; KHNMCOH 2019-01-003-003). The study was registered with the Clinical Research Information Service (CRIS) (KCT0003405, registered on 24 December 2018).

Before enrollment in the study, all subjects provided written informed consent and were evaluated for eligibility. The inclusion criteria for participants with FD were as follows: 1) aged between 19 and 75 years; 2) met the Rome IV criteria for FD (Stanghellini et al., 2016); 3) had an overall dyspeptic symptom score on a visual analog scale (VAS) of 0–100 points ≥40; 4) agreed not to receive any other treatment for dyspepsia during the study period; and 5) voluntarily agreed to participate and signed the informed consent form.

The exclusion criteria were as follows: 1) had an organic cause of dyspepsia such as peptic ulcer, gastroesophageal cancer, or biliary pancreatic disease within the last 1 year; 2) had obvious clinical symptoms of irritable bowel syndrome; 3) had alarm symptoms including weight loss, hematochezia, and dysphagia; 4) had psychiatric diseases or severe diseases associated with the heart, lung, liver, or kidney; 5) had a surgery history of the GI tract; 6) pregnancy or lactation; 7) took medications that affect the GI tract, such as prokinetics, PPIs, and anti-ulcer agents, non-steroidal anti-inflammatory drugs, anticholinergics, and steroids; 8) had participated in another clinical trial within less than 1 month before the randomization; 9) were positive for human immunodeficiency virus; 10) used alternative treatments including herbal medicine or acupuncture that may have an effect on indigestion; 11) could not cooperate or were unable to continuously participate in the clinical trial; 12) had blood clotting disorders, leukopenia, or seizure disorders; 13) had uncontrolled chronic diseases (e.g., hypertension and diabetes mellitus); 14) had allergies to the components of NHT or placebo; 15) had diseases that may affect the intake or absorption of drugs, such as dysphagia, glucose-galactose malabsorption, or lactase deficiency; and 16) had a history of alcohol abuse or drug dependence.

We collected the demographic information and checked the clinical history of each participant, including sex, age, weight, alcohol/smoking status, and *H. pylori* infection status.

### 2.3 Intervention

In this clinical trial, NHT and placebo were prescribed to treat patients with FD. Botanical drugs for the preparation of the NHT extract granules were purchased from Humanherb (Daegu, Korea), Omniherb (Yeongcheon, Korea), and Dongil Korean Pharmaceutical Co., (Daegu, Korea). Brown granules of the NHT herbal extract and placebo were produced by the National Institute for Korean Medicine Development (Gyeongsan, Republic of Korea) and Shinhwa Pharmaceutical Corporation (Daegu, Republic of Korea). Both facilities followed the strict guidelines of Korean Good Manufacturing Practice (KGMP) during production. Additionally, the botanical drugs used in the manufacturing of the trial drugs, preparation procedures, and quality management were rigorously quality controlled according to the standards set by the Korean Pharmacopoeia (KP) and Korean Herbal Pharmacopoeia (KHP).

Both NHT and placebo were administered in the form of granules. NHT contains the following 19 botanical drugs (dosage per serving): the dried ripe fruit of *Crataegus pinnatifida* Bunge (*Rosaceae*; Crataegi Fructus) (2.50 g); the stir-baked dried germinated ripe fruit of *Hordeum vulgare* L. (*Poaceae*; Hordei Fructus Germinatus) (2.50 g); the dried stem bark, root bark, or branch bark of *Magnolia officinalis* Rehder & E.H.Wilson (*Magnoliaceae*; Magnoliae Cortex) (1.88 g); the dried ripe pericarp of *Citrus reticulata* Blanco (*Rutaceae*; Citri Unshius Pericarpium) (1.88 g); the dried tuber of *Alisma plantago-aquatica* subsp. *orientale* (Sam.) Sam. (*Alismataceae*; Alismatis Rhizoma) (1.88 g); the dried rhizome of *Atractylodes lancea* (Thunb.) DC. (*Compositae*; Atractylodis Rhizoma) (1.25 g); the dried rhizome of *Cyperus rotundus* L. (*Cyperaceae*; Cyperi Rhizoma) (1.25 g); the dried unripe fruit of *Citrus trifoliata* L. (*Rutaceae*; Ponciri Fructus Immaturus) (1.25 g); the dried tuber of *Pinellia ternata* (Thunb.) Makino (*Araceae*; Pinelliae Tuber) (1.25 g); the dried sclerotium of *Poria cocos* (Schw.) Wolf (*Polyporaceae*; Poria Sclerotium) (1.25 g); the stir-baked fermented mixture (Massa Medicata Fermentata) (1.25 g) composed of six botanical drugs (the caryopsis of *Triticum aestivum* L. (*Poaceae*; Tritici Fructus Levis), the seed of *Vigna umbellata* (Thunb.) Ohwi and H. Ohashi (*Fabaceae*; Vignae Angularis Semen), the seed of *Prunus armeniaca* L. (*Rosaceae*; Armeniacae Semen), the stem and leaf of *Xanthium strumarium* L. (*Compositae*; Xanthii Fructus), the stem and leaf of *Artemisia annua* L. (*Compositae*; Artemisiae Annuae Herba), and the stem and leaf of *Persicaria hydropiper* (L.) Delarbre (*Polygonaceae*; Polygoni Hydropiperis Herba); the dried ripe fruit of *Wurfbainia villosa* (Lour.) Škorničk. & A.D.Poulsen (*Zingiberaceae*; Amomi Fructus) (1.25 g); the dried rhizome of *Sparganium stoloniferum* (Buch.-Ham. ex Graebn.) Buch.-Ham. ex Juz. (*Typhaceae*; Spargarnii Rhizoma) (1.25 g); the dried rhizome of *Curcuma phaeocaulis* Valeton (*Zingiberaceae*; Curcumae Rhizoma) (1.25 g); the dried rhizome of *Zingiber officinale* Roscoe (*Zingiberaceae*; Zingiberis Rhizoma) (1.25 g); the fresh rhizome of *Z. officinale* Roscoe (*Zingiberaceae*; Zingiberis Rhizoma Recens) (1.25 g); the dried aerial part of *Agastache rugosa* (Fisch. & C.A.Mey.) Kuntze (*Lamiaceae*; Agastachis Herba) (1.00 g); the dried root of *Aucklandia lappa* DC. (*Compositae*; Aucklandiae Radix) (0.67 g); and the dried root and rhizome of *Glycyrrhiza uralensis* Fisch. (*Leguminosae*; Glycyrrhizae Radix et Rhizoma) (0.67 g).

Specifically, raw botanical drugs were weighed and boiled with purified water at 95°C–105°C for 3 h. After filtering the extract, the filtrate was concentrated in a decompression evaporator below 55°C with 650–700 mmHg, dried below 50°C with 650–700 mmHg, and pulverized at 1,800 RPM to obtain 5.0 g of dry extract. The extraction yield was 18.75%. The dry extract was then mixed with the excipients of *Zea mays* (corn) starch (0.6 g), powdered cellulose (1.4 g), and small amounts of purified water and was dried in granule form to obtain one package of 7.0 g NHT granules. Tests were conducted on raw botanical drugs and dried extracts for identification, purity (residual pesticides and heavy metals), and microbiological examination, and the contents and results were found to be suitable. A chemical identification test was conducted in accordance with the procedure of thin-layer chromatography. High-performance liquid chromatography with the diode-array detection method was used for quantitative analysis; the hesperidin content (C_28_H_34_O_15_:610.56) was ensured to be ≥11.5 mg and the glycyrrhizic acid content (C_42_H_62_O_16_:822.93) was maintained at ≥5.0 mg in 1 dose (1 package). The placebo granules (7.0 g) consisted of lactose hydrate (3.268 g), *Z. mays* starch (3.080 g), cacao color powder (0.249 g), sepia color powder (0.300 g), Food Red No. 40 (0.005 g), *Ssanghwa* flavor (0.098 g), and small amounts of purified water without any effective components for the treatment of FD. A content test was conducted to confirm that the inclusion of the main ingredients, including hesperidin and glycyrrhizic acid, clearly distinguished the investigational drug from the placebo. Finally, the NHT and placebo granules were packed separately in opaque aluminum bags and were indistinguishable in appearance. The random codes were labeled by an independent manufacturer. The clinical trial pharmacist at each center provided a packaged drug to the subjects based on random allocation. Both drugs were administered to the subjects three times a day at 30 min after each meal for 4 weeks.

### 2.4 Randomization and allocation concealment

A total of 116 patients with FD who met the eligible criteria were randomly assigned at a ratio of 1:1 into either the NHT or placebo group. For randomization, an independent statistician not directly involved in this study generated a set of 116 random numbers using the PROC PLAN in SAS (SAS Institute Inc., Cary, North Carolina, United States). To maintain the randomization process, random number tables for allocation were concealed using sealed opaque envelopes throughout the study period. The investigational drugs, packaged identically to conceal the intervention allocation for both the participants and investigators, were distributed equally to both study sites with label numbers from 1 to 58 at a ratio of 1:1. After enrollment, the subjects received the study number in the order of randomization and were assigned to the corresponding groups for 4 weeks and another 4 weeks for follow-up. All investigators and participants were blinded to treatment allocation throughout the study.

### 2.5 Outcome assessment

#### 2.5.1 Primary outcome measure

The total dyspepsia symptom (TDS) scale was the primary outcome measure to evaluate the severity and degree of improvement of overall dyspeptic symptoms in patients with FD after 4 weeks administration of NHT compared with placebo. It uses a 4-point Likert scale to assess eight different symptoms related to indigestion: epigastric pain, epigastric burning, postprandial fullness/bloating, early satiety, nausea, vomiting, belching, and other dyspeptic symptoms. The Likert scale ranges from 0 (none) to 3 (severe) and is used to rate the intensity of each symptom. The overall score was calculated by adding points for each item, with higher scores indicating more severe indigestion symptoms. The TDS questionnaire has been used as the primary outcome measure in several clinical trials on FD (Zhang et al., 2013; Ko et al., 2018). It was evaluated throughout the 4 weeks study period, including at follow-up visits.

#### 2.5.2 Secondary outcome measures

Regarding the secondary outcome variables, the single dyspepsia symptom (SDS) scale, FD-related quality of life (FD-QoL) questionnaire, *Damum* questionnaire (DQ), and food retention questionnaire (FRQ) were administered throughout the 4 weeks study period.

To evaluate the intensity of dyspeptic symptoms, the four main symptoms of FD on the SDS were measured using a 4-point Likert scale. Each symptom was assessed based on the frequency, intensity, and level of discomfort experienced by the patient (Zhang et al., 2013).

The FD-QoL questionnaire consists of 21 items measuring the QoL of patients with FD, grouped into four categories: diet, daily activity, emotion, and social functioning. These items were assessed using a 5-point Likert scale (Lee et al., 2006).

DQ is a diagnostic tool for identifying a phlegm pattern in TKM and is associated with symptoms such as indigestion, dizziness, and headache (Park et al., 2011). It comprises 14 items rated on a 7-point Likert scale. A higher DQ score indicates a greater probability of a phlegm pattern (Park et al., 2006).

The FRQ is used to diagnose a food retention pattern in TKM, which is characterized by symptoms such as abdominal fullness and pain (Zhu et al., 2002). It comprises 17 items rated on a 7-point Likert scale. Scores of 1, 2, 3, and 4 were converted to 0 points, whereas scores of 5, 6, and 7 were converted to 1 point. A total score of six or greater indicates that the individual has a food retention pattern (Park et al., 2013).

The overall treatment effect (OTE) (Van Zanten et al., 2006) was assessed to evaluate the improvement in symptoms by treatment as perceived by the patient at the visit after taking the investigational drugs (weeks 2 and 4). We re-evaluated the TDS and OTE scores to assess the continued impact of the intervention (week 8).

We used EGG (Yin and Chen, 2013) to measure the gastric myoelectrical activity (GMA) of patients with PDS or overlap subtypes of FD, both before and after treatment. Participants who had not eaten for more than 8 h and were lying on their backs had three active electrodes with gel applied to their epigastric skin. The evaluation of gastric motility was performed using multichannel EGG (Polygraf ID, Medtronic A/S, Denmark) in three stages: the measurements were recorded 30 min before and after a standard nutritional beverage was consumed. During the EGG assessment, measurements were performed for dominant frequency and power, percentage of normal/brady/tachygastria and arrhythmia, and power ratio to assess GMA.

The evaluation of the 36-item standard tool for pattern identification of FD (PIFD) was performed for patients in the screening visit to classify the pattern of disease according to the TKM theory and determine the impact on treatment effectiveness: 1) Spleen and stomach deficiency with cold pattern (SSDC); 2) Spleen deficiency with *qi* stagnation pattern (SDQS); 3) Disharmony of liver and stomach systems pattern (DLSS); 4) Tangled cold and heat pattern (TACH); 5) Dampness and heat in the spleen and stomach systems pattern (DHSS); and 6) Food retention disorder pattern (FRED) (Ha, 2020).

### 2.6 Safety and adverse event monitoring

To evaluate the safety of the intervention, vital signs were checked at each visit, and electrocardiography and laboratory tests, including blood tests and urinalysis, were performed before and after the administration of the investigational drugs. We monitored any negative side effects that occurred throughout the study period.

### 2.7 Statistical analysis

Outcome analysis was performed by an independent statistician using SPSS software (version 25.0; IBM SPSS Statistics, Armonk, New, United States) in a blinded manner. The effectiveness of the NHT was evaluated using the full analysis set (FAS), which included all participants who were randomly assigned, received the investigational products, and were subsequently assessed at least once. In instances of missing values for the primary variables, the last observation carried forward method was used as a substitute. The safety of the drug was assessed in randomly assigned participants who took the investigational drugs at least once. Quantitative variables are presented as the mean ± standard deviation (SD), and qualitative variables are presented as numbers and percentages. Pearson’s chi-square test or Fisher’s exact test was used for discrete variables, whereas the independent two-sample *t*-test or Mann–Whitney *U* test was used for continuous data. The assessment of the therapeutic effects of the drug compared to that at baseline was performed using the paired sample *t*-test or Wilcoxon signed-rank test and repeated measures analysis. For safety assessment, the frequency of adverse events and clinically significant changes in laboratory measurements were compared between the two groups. A *p* < 0.05 was considered statistically significant.

Subgroup analyses were performed for FD patients with subtypes, including sex, age, body mass index (BMI), FD subtype, and PIFD, based on the degree of improvement on the TDS scale after 4 weeks treatment.

## 3 Results

### 3.1 Demographic and clinical characteristics of the participants

**FIGURE 1:**
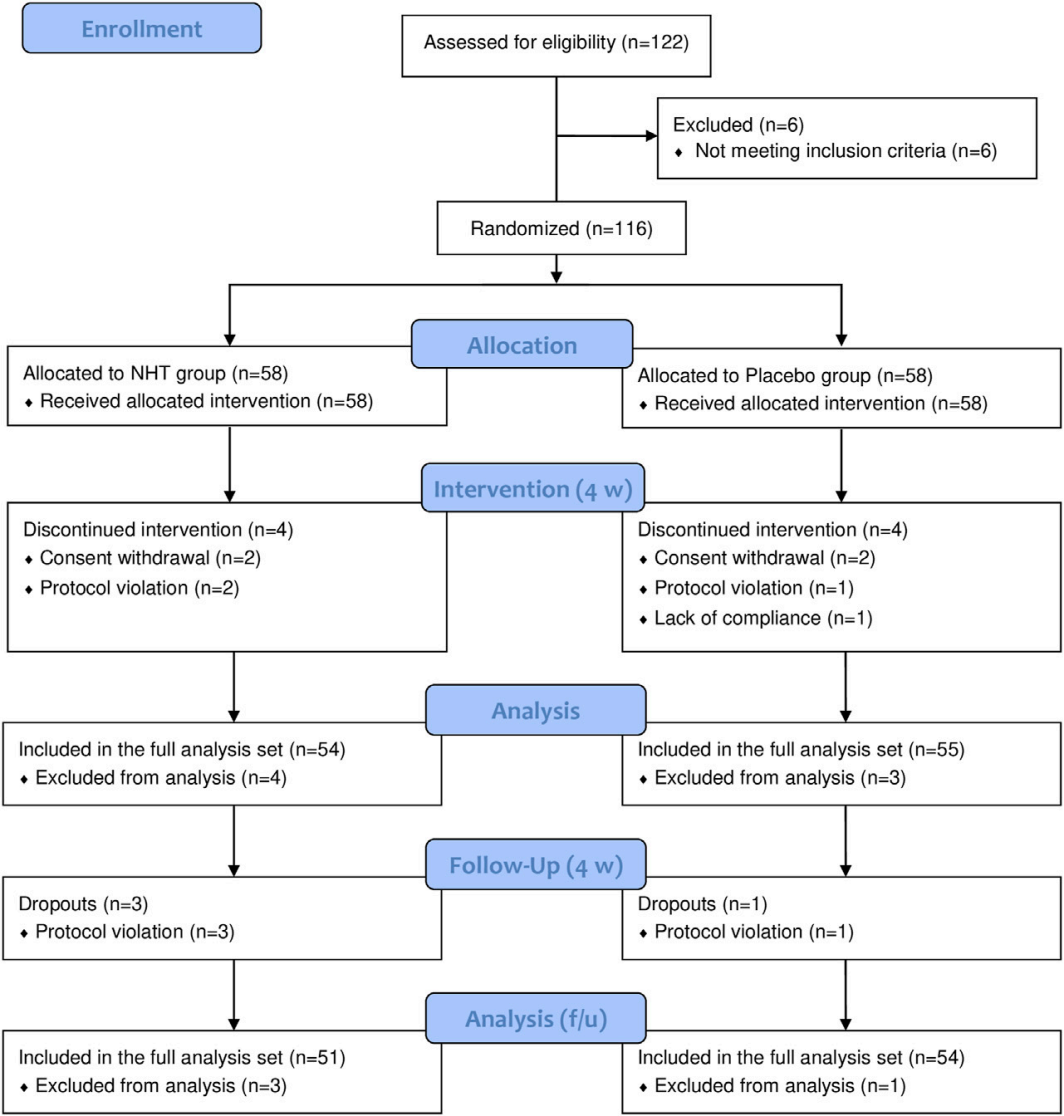
Flow chart of the trial; NHT, *Naesohwajung-tang*.

From 10 May 2019, to 1 September 2020, 122 patients were recruited to participate in this study. Six participants were excluded as they did not meet the inclusion criteria. A total of 116 patients with FD were enrolled from two sites and randomly assigned to either the NHT group (*n* = 58) or placebo group (*n* = 58). During the 4 weeks trial period, seven patients were removed from the efficacy assessment owing to issues with consent and violations of the study protocol that occurred after randomization. Thus, 109 patients (54 in the NHT group and 55 in the placebo group) were included in the FAS at week 4. By the follow-up period of 4 weeks after the end of drug administration, three patients in the NHT group and one in the placebo group dropped out due to protocol violation. The number of patients who completed the trial in the NHT and placebo groups was 51 and 53, respectively. One subject in the placebo group dropped out with less than 70% compliance, but was included in the FAS. The detailed research flow and reasons for dropping out are presented in Figure 1 (Schulz et al., 2010).

The demographic characteristics of the patients at baseline were clinically similar between the two groups, including mean age, sex distribution, and mean BMI (Table 2). No clinically significant differences were observed between the two groups in the evaluation variables such as the VAS for dyspeptic symptoms, TDS scale, SDS scale, and FD-QoL questionnaire at baseline.

**TABLE 2:** Baseline clinical characteristics of the patients included in the NHT and placebo groups.

Characteristics	NHT	Placebo	*p*-value
Number	58	58	—
Mean age (years)	47.81 ± 13.12	47.57 ± 13.63	0.923^a^
Sex, *n* (M/F)	13/45	17/41	0.396^b^
Mean weight (kg)	56.87 ± 10.99	60.44 ± 10.90	0.078^c^
Mean BMI (kg/m^2^)	21.56 ± 3.35	22.61 ± 3.14	0.082^a^
Current alcohol use, *n* (%)	11 (19.0)	19 (32.8)	0.090^b^
Current smoking, *n* (%)	5 (8.6)	5 (8.6)	1.000^b^
*Helicobacter pylori* infection, *n* (%)	5 (8.6)	2 (3.4)	0.463^d^
VAS for overall dyspeptic symptoms	67.90 ± 15.05	64.93 ± 12.41	0.249^a^
TDS scale	10.57 ± 4.11	9.97 ± 3.66	0.405^a^
SDS scale			
Epigastric pain	3.36 ± 2.50	3.48 ± 2.48	0.850^c^
Epigastric burning	3.88 ± 1.96	3.10 ± 2.28	0.058^c^
Postprandial fullness	5.95 ± 2.39	5.79 ± 2.03	0.638^c^
Early satiation	4.60 ± 2.87	3.91 ± 2.61	0.200^c^
FD-QoL questionnaire	30.91 ± 18.71	28.34 ± 15.96	0.568^c^

^a^ Independent two-sample *t*-test.

^b^ Pearson’s chi-squared test.

^c^ Mann–Whitney *U* test.

^d^ Fisher’s exact test.

BMI, body mass index; FD-QoL, functional dyspepsia-related quality of life; NHT, *Naesohwajung-tang*; SDS, single dyspepsia symptom; TDS, total dyspepsia symptom; VAS, visual analog scale. Baseline values were analyzed using Pearson’s chi-squared or Fisher’s exact tests for categorical variables and the independent two-sample *t*-test or Mann–Whitney *U* test for continuous variables. Continuous values are presented as the mean ± SD. *p* < 0.05 indicates statistically significant differences between the NHT and placebo groups.

### 3.2 Clinical outcomes

#### 3.2.1 Primary outcome evaluation for dyspeptic symptoms

**FIGURE 2:**
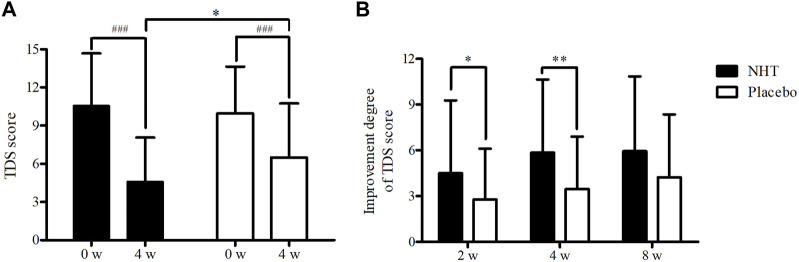
**(A)** Comparison of the score of the TDS scale. The Mann–Whitney *U* test was used to compare the two groups (*p < 0.05), and the repeated measures ANOVA was used for comparison with the baseline value (^###^ *p* < 0.001). **(B)** Comparison of the improvement degree of the TDS scale score. The independent two-sample *t*-test was used to compare two groups (^*^ *p* < 0.05; ^**^ *p* < 0.01); NHT, *Naesohwajung-tang*; TDS, total dyspepsia symptom.

The changes in the TDS scores before and after treatment and the degree of improvement are described in Tables 3, 4, respectively. Both groups showed significant improvements in dyspeptic symptoms related to TDS at week 4 compared to baseline as well as at the 2 and 8 weeks follow-ups. The mean baseline TDS scores were not statistically different between the NHT (10.57 ± 4.11) and control groups (9.97 ± 3.66). After 4 weeks treatment, the TDS score of the NHT group was significantly lower than that of the placebo group (4.59 ± 3.48 vs. 6.49 ± 4.25, *p* = 0.011) (Figure 2A), whereas the differences between the results at weeks 2 and 8 were not significant. In addition, the improvement degree of the TDS score were significantly higher in the NHT group than in the placebo group after the 2 and 4 weeks treatments (4.50 ± 4.77 vs. 2.79 ± 3.32, *p* = 0.030; 5.85 ± 4.80 vs. 3.45 ± 3.45, *p* = 0.004) (Figure 2B). At the 8 weeks follow-up, 4 weeks after taking the medication, the improvement in the TDS score did not differ between the two groups.

**TABLE 3:** Analysis and comparison of the scores of the TDS scale, OTE, SDS scale, FD-QoL questionnaire, and DQ, and FR proportion at baseline and post-treatment between the NHT and placebo groups.

Variables	Weeks	Mean ± SD (*n*)				*p*-value
		NHT		Placebo		
TDS scale	0	10.57 ± 4.11	(58)	9.97 ± 3.66	(58)	0.405^a^
	2	6.05 ± 3.61^###^	(56)	7.14 ± 4.31^###^	(57)	0.137^b^
	4	4.59 ± 3.48^###^	(54)	6.49 ± 4.25^###^	(55)	0.011^b^ ^,^*
	8	4.53 ± 3.32^###^	(51)	5.72 ± 4.15^###^	(54)	0.137^b^
OTE	2	2.04 ± 1.53	(56)	1.30 ± 1.96	(57)	0.016^b^ ^,^*
	4	2.89 ± 1.91^##^	(54)	1.62 ± 2.35	(55)	0.002^b^ ^,^**
	8	2.80 ± 2.58	(51)	1.28 ± 2.01	(54)	0.000^b^ ^,^***
SDS scale						
Epigastric pain	0	3.36 ± 2.50	(58)	3.48 ± 2.48	(58)	0.850^b^
	2	2.11 ± 2.33^##^	(56)	1.91 ± 2.27^###^	(57)	0.569^b^
	4	1.59 ± 2.12^###^	(54)	1.56 ± 2.25^###^	(55)	0.734^b^
Epigastric burning	0	3.88 ± 1.96	(58)	3.10 ± 2.28	(58)	0.058^b^
	2	2.20 ± 1.80^###^	(56)	2.14 ± 2.33^##^	(57)	0.631^b^
	4	1.50 ± 1.93^###^	(54)	1.89 ± 2.20^##^	(55)	0.451^b^
Postprandial fullness	0	5.95 ± 2.39	(58)	5.79 ± 2.03	(58)	0.638^b^
	2	3.84 ± 2.04^###^	(56)	4.58 ± 2.56^##^	(57)	0.232^b^
	4	3.28 ± 2.32^###^	(54)	4.16 ± 2.68^###^	(55)	0.127^b^
Early satiation	0	4.60 ± 2.87	(58)	3.91 ± 2.61	(58)	0.200^b^
	2	2.36 ± 2.28^###^	(56)	3.07 ± 2.33	(57)	0.142^b^
	4	1.81 ± 2.06^###^	(54)	2.71 ± 2.47^##^	(55)	0.060^b^
FD-QoL questionnaire	0	30.91 ± 18.71	(58)	28.34 ± 15.96	(58)	0.568^b^
	2	20.39 ± 16.34^###^	(56)	23.33 ± 17.91^##^	(57)	0.333^b^
	4	16.59 ± 14.82^###^	(54)	21.85 ± 18.87^###^	(55)	0.131^b^
DQ	0	10.07 ± 3.35	(58)	8.99 ± 3.42	(58)	0.091^a^
	2	7.67 ± 4.27^###^	(56)	7.62 ± 3.74^#^	(57)	0.953^a^
	4	6.20 ± 3.46^###^	(54)	7.40 ± 4.01^#^	(55)	0.096^a^
FR, *n* (%)	0	37 (63.8)	(58)	37 (63.8)	(58)	1.000^d^
	2	23 (41.1)^c^ ^,##^	(56)	28 (49.1)^c^ ^,#^	(57)	0.390^d^
	4	16 (29.6)^c^ ^,###^	(54)	25 (45.5)^c^ ^,#^	(55)	0.088^d^

^a^ Independent two-sample *t*-test.

^b^ Mann–Whitney *U* test.

^c^ McNemar’s test.

^d^ Pearson’s chi-squared test.

DQ, *Damum* questionnaire; FD-QoL, functional dyspepsia-related quality of life; FR, food retention; NHT, *Naesohwajung-tang*; OTE, overall treatment effect; SDS, single dyspepsia symptom; SD, standard deviation; TDS, total dyspepsia symptom. Pearson’s chi-squared test for categorical variables and the independent two-sample *t*-test or Mann–Whitney *U* test for continuous variables were used. McNemar’s test was used to compare the proportion of patients diagnosed with FR before and after intervention. The significant differences compared to the baseline value were analyzed using repeated measures ANOVA. Continuous values are presented as the mean ± SD. *p < 0.05, **p < 0.01, and ***p < 0.001 indicate statistically significant differences between the NHT and placebo groups. ^#^ *p* < 0.05, ^##^ *p* < 0.01, and ^###^ *p* < 0.001 indicate statistically significant differences compared to the baseline value (week 0).

**TABLE 4:** Analysis and comparison of the improvement degree on the TDS scale, OTE, SDS scale, FD-QoL questionnaire, and DQ after treatments between the NHT and placebo groups.

Variables	Weeks	Mean ± SD (*n*)				*p*-value
		NHT		Placebo		
TDS scale	2	4.50 ± 4.77	(56)	2.79 ± 3.32	(57)	0.030^a^ ^,^*
	4	5.85 ± 4.80	(54)	3.45 ± 3.45	(55)	0.004^a^ ^,^**
	8	5.94 ± 4.90	(51)	4.24 ± 4.12	(54)	0.057^a^
OTE	4	0.85 ± 1.63	(54)	0.35 ± 1.54	(55)	0.173^b^
	8	0.73 ± 2.55	(51)	0.00 ± 1.78	(54)	0.020^b^ ^,^*
SDS scale						
Epigastric pain	2	1.18 ± 2.71	(56)	1.58 ± 2.13	(57)	0.456^b^
	4	1.65 ± 2.44	(54)	1.91 ± 2.54	(55)	0.738^b^
Epigastric burning	2	1.82 ± 2.25	(56)	0.91 ± 1.91	(57)	0.016^b^ ^,^*
	4	2.44 ± 2.18	(54)	1.07 ± 2.22	(55)	0.001^b^ ^,^*
Postprandial fullness	2	2.13 ± 2.11	(56)	1.21 ± 2.63	(57)	0.057^b^
	4	2.69 ± 2.38	(54)	1.65 ± 2.43	(55)	0.048^b^ ^,^*
Early satiation	2	2.25 ± 2.87	(56)	0.84 ± 2.58	(57)	0.021^b^ ^,^*
	4	2.80 ± 2.73	(54)	1.20 ± 2.65	(55)	0.004^b^ ^,^**
FD-QoL questionnaire	2	10.20 ± 13.79	(56)	5.14 ± 9.32	(57)	0.025^a^ ^,^*
	4	14.17 ± 16.00	(54)	6.60 ± 9.76	(55)	0.032^b^ ^,^*
DQ	2	2.35 ± 3.56	(56)	1.41 ± 3.56	(57)	0.162^a^
	4	3.83 ± 3.92	(54)	1.65 ± 3.96	(55)	0.005^a^ ^,^**

^a^ Independent two-sample *t*-test.

^b^ Mann–Whitney *U* test.

DQ, *Damum* questionnaire; FD-QoL, functional dyspepsia-related quality of life; NHT, *Naesohwajung-tang*; OTE, overall treatment effect; SDS, single dyspepsia symptom; SD, standard deviation; TDS, total dyspepsia symptom. Statistically significant differences between the two groups were analyzed using the independent two-sample *t*-test or Mann–Whitney *U* test. Continuous values are presented as the mean ± SD. *p < 0.05 and **p < 0.01 indicate statistically significant differences between the NHT and placebo groups.

#### 3.2.2 Secondary outcome evaluation for dyspeptic symptoms

**FIGURE 3:**
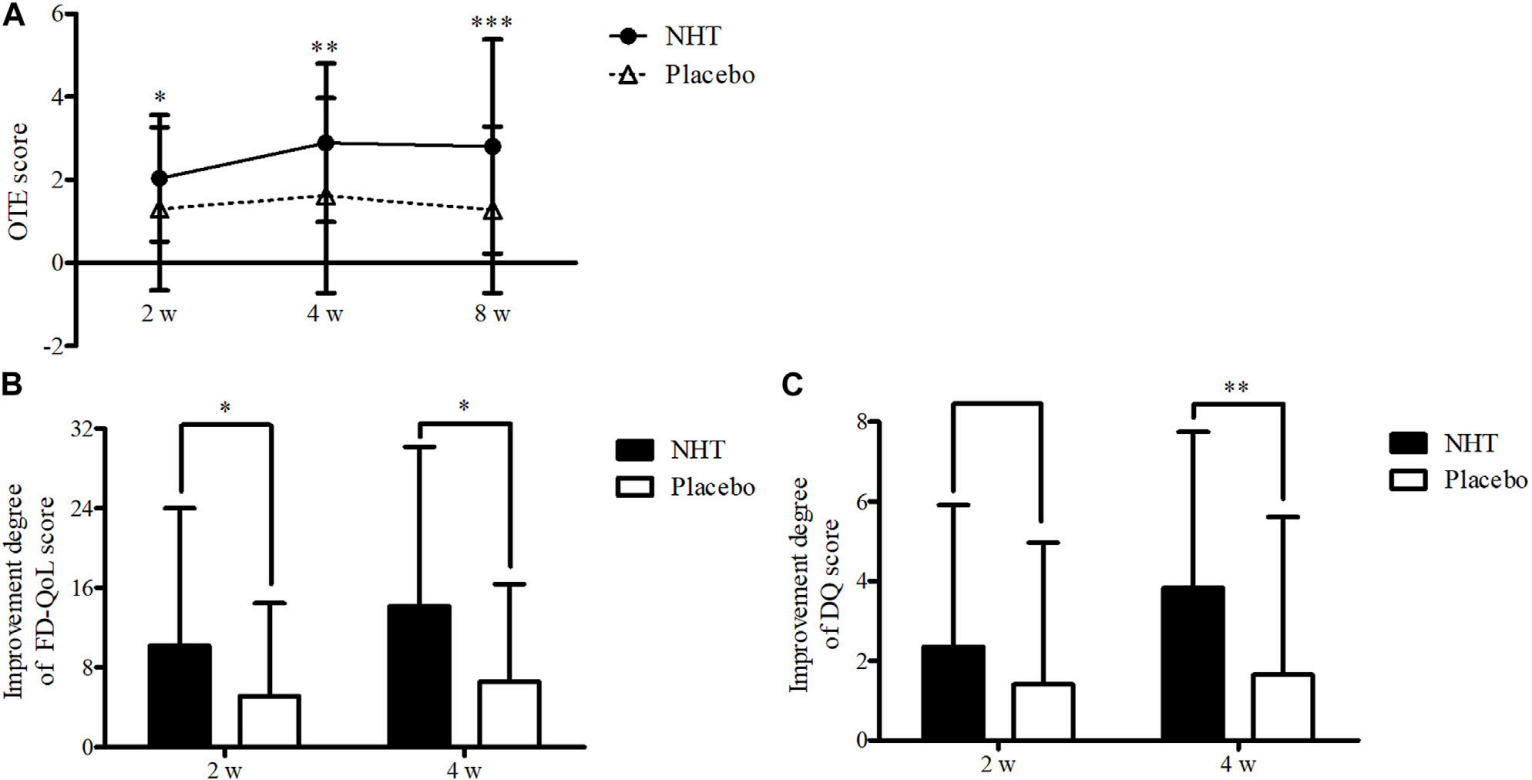
**(A)** Comparison of the OTE score. The Mann–Whitney *U* test was used to compare two groups (*p < 0.05, **p < 0.01, and ***p < 0.001). **(B)** Comparison of the improvement degree in the scores of the FD-QoL questionnaire. The independent two-sample *t*-test or Mann–Whitney *U* test was used to compare two groups (*p < 0.05). **(C)** Comparison of the improvement degree in the scores of the DQ. The independent two-sample *t*-test was used to compare two groups (**p < 0.01); DQ, *Damum* questionnaire; FD-QoL, functional dyspepsia-related quality of life; NHT, *Naesohwajung-tang*; OTE, overall treatment effect.

Table 3 shows the pre-treatment and post-treatment scores of the OTE, SDS scale, FD-QoL questionnaire, DQ, and the proportion of FR diagnosis for each group in relation to the time from enrollment to follow-up. In both groups, epigastric pain, epigastric burning, postprandial fullness, and early satiation scores, as measured by the SDS scale, significantly decreased after the 2 and 4 weeks treatments, as well as the FD-QoL and DQ scores. However, the 2 weeks score of early satiation in the placebo group did not show any significant changes. The OTE score in the NHT group significantly increased after the 4 weeks treatment compared with that after the 2 weeks treatment (*p* < 0.01), whereas there were no significant differences within the placebo group. When comparing the differences between the two groups at each time point, OTE values measured at weeks 2, 4, and 8 were significantly higher in the NHT group than in the placebo group (2.04 ± 1.53 vs. 1.30 ± 1.96, *p* = 0.016; 2.89 ± 1.91 vs. 1.62 ± 2.35, *p* = 0.002; 2.80 ± 2.58 vs. 1.28 ± 2.01, *p* = 0.000) (Figure 3A). After the 2 and 4 weeks treatments, there were no significant differences in the other scales between the two groups.

**FIGURE 4:**
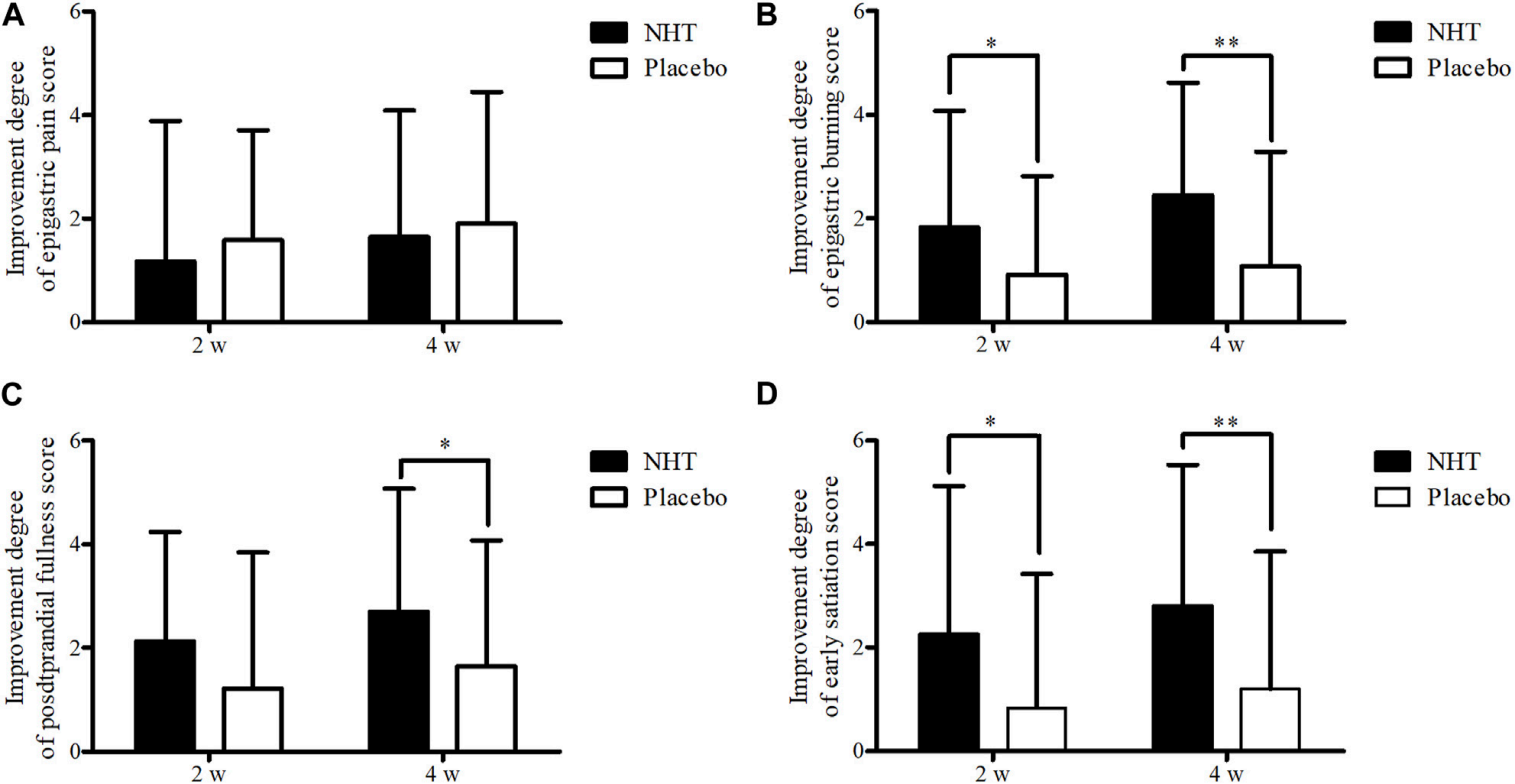
Comparison of the degree of improvement of the score on the SDS scale. The Mann–Whitney *U* test was used to compare two groups (*p < 0.05 and **p < 0.01). **(A)** Epigastric pain. **(B)** Epigastric burning. **(C)** Postprandial fullness. **(D)** Early satiation; SDS, single dyspepsia symptom; NHT, *Naesohwajung-tang*.

The degrees of improvement in the OTE, SDS, FD-QoL, and DQ scores after treatment in each group are presented in Table 4. Regarding the OTE score, in the 8 weeks observation time, significant differences were observed between the two groups (0.73 ± 2.55 vs. 0.00 ± 1.78, *p* = 0.020). The NHT group had a greater degree of improvement compared to the placebo group for the scores of epigastric burning (1.82 ± 2.25 vs. 0.91 ± 1.91, *p* = 0.016; 2.44 ± 2.18 vs. 1.07 ± 2.22, *p* = 0.001) (Figure 4B), early satiation (2.25 ± 2.87 vs. 0.84 ± 2.58, *p* = 0.021; 2.80 ± 2.73 vs. 1.20 ± 2.65, *p* = 0.004), (Figure 4D), and FD-QoL (10.20 ± 13.79 vs. 5.14 ± 9.32, *p* = 0.025; 14.17 ± 16.00 vs. 6.60 ± 9.76, *p* = 0.032) (Figure 3B) after 2 and 4 weeks of treatment. Additionally, the NHT group had a greater degree of improvement in postprandial fullness (2.69 ± 2.38 vs. 1.65 ± 2.43, *p* = 0.048) (Figure 4C) and DQ (3.83 ± 3.92 vs. 1.65 ± 3.96, *p* = 0.005) (Figure 3C) after 4 weeks of treatment. However, no significant differences were observed in the degree of improvement in the epigastric pain scores (Figure 4A) between the two groups after 2 and 4 weeks of treatment.

In both groups, the proportions of FR significantly decreased after the 2 and 4 weeks treatments compared with those before treatment, with no significant difference between the two groups.

#### 3.2.3 Secondary outcome evaluation for gastric myoelectrical activity (GMA)

**FIGURE 5:**
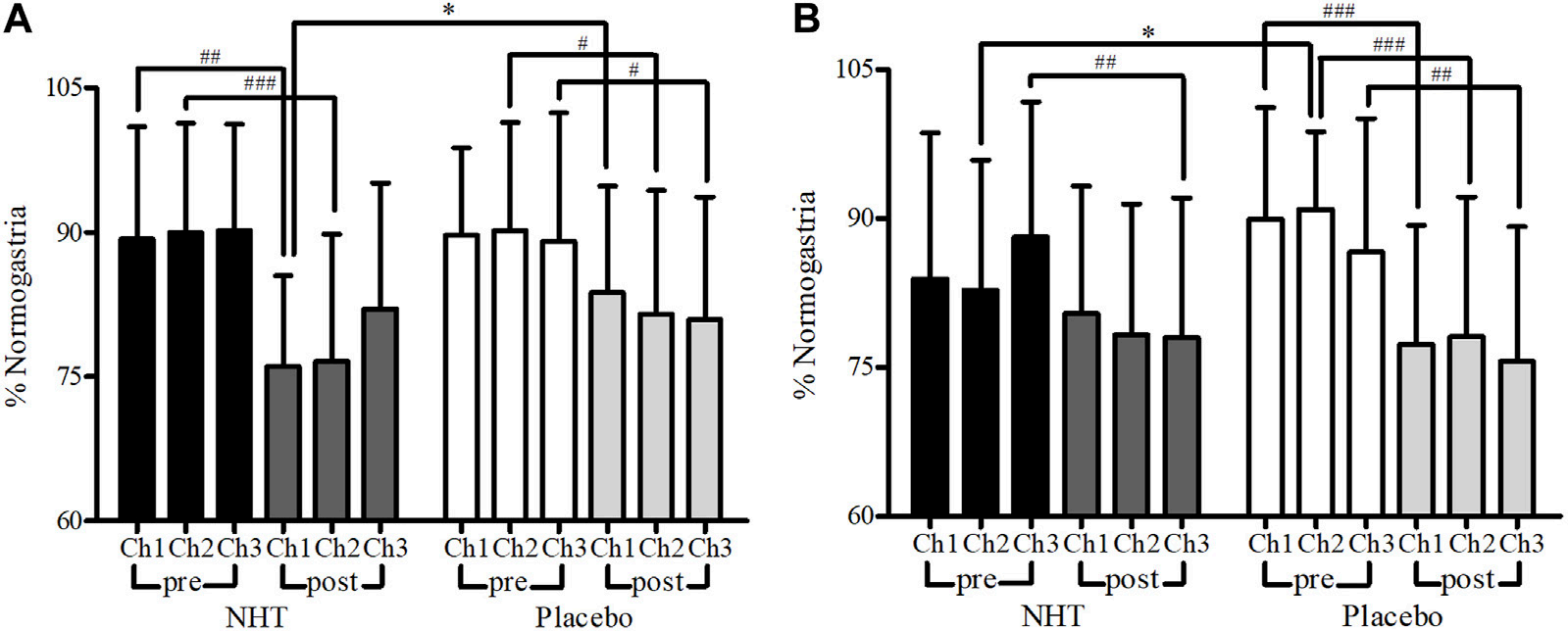
Comparison of percentage of normogastria measured by EGG recording. The independent two-sample *t*-test or Mann–Whitney *U* test was used to compare two groups (*p < 0.05), and a paired sample *t*-test was used to compare preprandial and postprandial values (^#^ *p* < 0.05, ^##^ *p* < 0.01, and ^###^ *p* < 0.001). **(A)** Baseline. **(B)** After treatment (week 4); Ch, channel; EGG, electrogastrography; NHT, *Naesohwajung-tang*.

The efficacy of NHT on GMA was evaluated using a surface multichannel EGG to detect the abnormal gastric motility for participants classified as PDS or overlap subtype. The EGG parameters and values evaluated at baseline and week 4 are presented in Supplementary Tables S1, S2. Among a total of 84 patients with FD whose GMA was assessed by EGG at baseline (44 in the NHT group and 40 in the placebo group), 47 had some missing data that could not be used for the analysis (21 in the NHT group and 26 in the placebo group); thus, EGG results of a total of 37 subjects were included in the outcome analysis. At baseline, there were two significant differences of GMA parameters in the postprandial recording between the two groups: percentage of normogastria at Ch1 (76.04 ± 9.41 vs. 83.71 ± 11.09, *p* = 0.029) and percentage of tachygastria at Ch1 (7.34 ± 5.29 vs. 3.14 ± 3.13, *p* = 0.022). Comparing the values before and after meals in the NHT group, we observed a significant decrease in the percentage of normogastria in Ch1 and Ch2, a significant increase in the percentage of bradygastria in Ch2 and Ch3, a significant increase in the percentage of tachygastria in all channels, and a significant increase in the percentage of arrhythmia in Ch1 and Ch2. In contrast, in the placebo group, a significant decrease in the percentage of normogastria in Ch2 and Ch3, a significant increase in the percentage of bradygastria in Ch2, and a significant increase in the percentage of tachygastria in all channels were observed (Figure 5A). For all channels, the power ratio was found to be 1 or higher, and there was no significant difference between the two groups.

Among the 79 patients with FD (41 in the NHT group and 38 in the placebo group) who underwent EGG testing after 4 weeks of treatment, EGG results were analyzed for a total of 50 participants, excluding 29 subjects with detected missing values (17 in the NHT group and 12 in the placebo group). At 4 weeks recoding, the differences in GMA values measured in preprandial were observed in the percentage of normogastria in Ch2 (82.80 ± 13.07 vs. 90.92 ± 7.81, *p* = 0.012) and percentage of tachygastria in Ch2 (5.56 ± 9.20 vs. 1.66 ± 3.79, *p* = 0.040). When the preprandial and postprandial values were compared, the NHT group showed a significant decrease in the percentage of normogastria in Ch3, a significant increase in the percentage of bradygastria in all channels, and a significant increase in the percentage of tachygastria in Ch3. In contrast, in the placebo group, a significant decrease in the percentage of normogastria in all channels, a significant increase in the percentage of bradygastria in all channels, an increase in the percentage of tachygastria in Ch1 and Ch2, and a significant increase in the percentage of arrhythmia in Ch1 were observed (Figure 5B). Similarly, the power ratio was found to be 1 or higher in all channels, and there was no significant difference between the two groups.

#### 3.2.4 Subgroup analyses of the improvement degree in the scores of TDS scale

**FIGURE 6:**
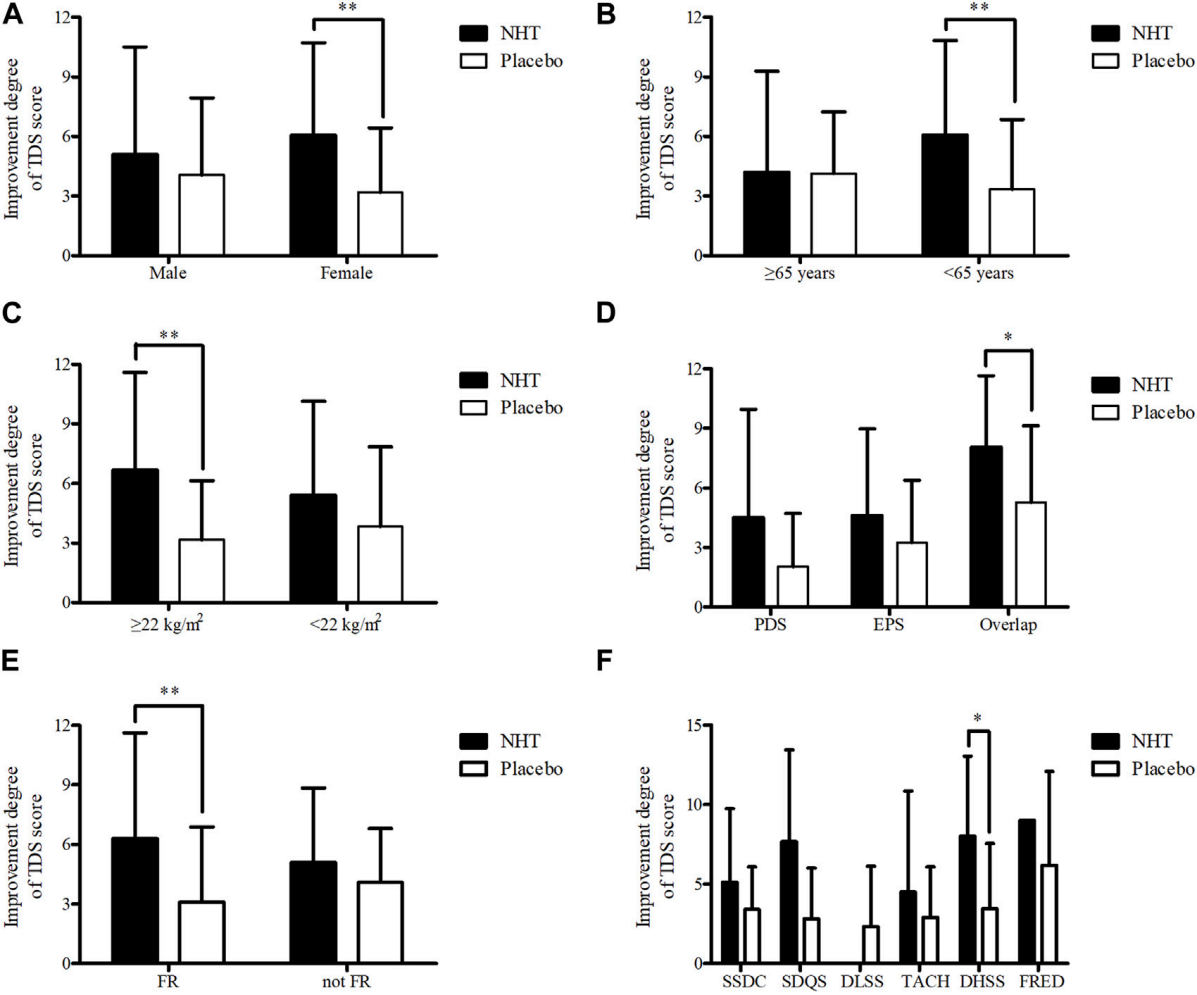
Subgroup analyses of the improvement degree of score on the TDS scale. The independent two-sample *t*-test or Mann–Whitney *U* test was used to compare two groups (*p < 0.05 and **p < 0.01). **(A)** Sex. **(B)** Age. **(C)** BMI. **(D)** FD subtype. **(E)** FR diagnosis. **(F)** PIFD; BMI, body mass index; DHSS, Dampness and heat in the spleen and stomach systems pattern; DLSS, Disharmony of liver and stomach systems pattern; EPS, epigastric pain syndrome; FR, food retention; FRED, Food retention disorder pattern; NHT, *Naesohwajung-tang*; PDS, postprandial distress syndrome; PIFD, pattern identification of functional dyspepsia; SDQS, Spleen deficiency with *qi* stagnation pattern; SSDC, Spleen and stomach deficiency with cold pattern; TACH, Tangled cold and heat pattern; TDS, total dyspepsia symptom.

Subgroup analyses of the changes in the TDS scale scores before and after the 4 weeks treatment were performed based on sex (male or female), age (≥65 as elderly population or <65 years), BMI (≥22 kg/m^2^ or <22 kg/m^2^ as the mean value of all subjects), FD subtypes (PDS, EPS, or overlap), FR diagnosis (FR or not FD), and PIFD (SSDC, SDQS, DLSS, TACH, DHSS, or FRED) (Table 5). The NHT group had a significantly higher degree of improvement in TDS scores for female patients compared to that of the placebo group (6.05 ± 4.68 vs. 3.18 ± 3.25, *p* = 0.002) (Figure 6A). In patients under 65, the improvement degree of the TDS scores was significantly higher in the NHT group than in the placebo group (6.06 ± 4.77 vs. 3.34 ± 3.52, *p* = 0.002) (Figure 6B). Patients with BMI ≥ 22 showed better TDS score improvement in the NHT group compared to the placebo (6.68 ± 4.91 vs. 3.16 ± 2.98, *p* = 0.006) (Figure 6C). For the overlap subtype of FD patients, the improvement degree of the TDS scores was significantly higher in the NHT group than in the placebo group (8.05 ± 3.61 vs. 5.28 ± 3.85, *p* = 0.028) (Figure 6D). FD patients diagnosed with FR had higher TDS improvement in the NHT group than in the placebo (6.29 ± 5.33 vs. 3.09 ± 3.79, *p* = 0.006) (Figure 6E). FD patients classified as the DHSS group showed better TDS score improvement in the NHT group than in the placebo (8.00 ± 5.06 vs. 3.44 ± 4.13, *p* = 0.048) (Figure 6F).

**TABLE 5:** Subgroup analyses of comparing the effect of NHT to placebo on the improvement degree in the TDS scale after 4 weeks treatment.

Subgroup	Mean ± SD (*n*)				*p*-value
	NHT		Placebo		
Improvement degree of score in TDS scale	5.85 ± 4.80	(54)	3.45 ± 3.45	(55)	0.004^a^ ^,^**
Sex (male)	5.09 ± 5.41	(11)	4.06 ± 3.88	(17)	0.378^b^
Sex (female)	6.05 ± 4.68	(43)	3.18 ± 3.25	(38)	0.002^b^ ^,^**
Age (≥65 years)	4.17 ± 5.12	(6)	4.13 ± 3.09	(8)	0.985^a^
Age (<65 years)	6.06 ± 4.77	(47)	3.34 ± 3.52	(48)	0.002^b^ ^,^**
BMI (≥22 kg/m^2^)	6.68 ± 4.91	(19)	3.16 ± 2.98	(31)	0.006^b^ ^,^**
BMI (<22 kg/m^2^)	5.40 ± 4.75	(35)	3.83 ± 4.01	(24)	0.191^a^
PDS	4.52 ± 5.44	(21)	2.05 ± 2.66	(21)	0.071^a^
EPS	4.62 ± 4.37	(13)	3.25 ± 3.13	(16)	0.336^a^
Overlap	8.05 ± 3.61	(20)	5.28 ± 3.85	(18)	0.028^a^ ^,^*
FR	6.29 ± 5.33	(34)	3.09 ± 3.79	(35)	0.006^a^ ^,^**
Not FR	5.10 ± 3.74	(20)	4.10 ± 2.71	(20)	0.340^a^
SSDC	5.13 ± 4.63	(38)	3.41 ± 2.67	(22)	0.073^a^
SDQS	7.67 ± 5.77	(3)	2.83 ± 3.19	(6)	0.262^b^
DLSS	—	(0)	2.33 ± 3.79	(3)	—
TACH	4.50 ± 6.36	(2)	2.90 ± 3.18	(10)	0.758^b^
DHSS	8.00 ± 5.06	(10)	3.44 ± 4.13	(9)	0.048^a^ ^,^*
FRED	9.00	(1)	6.20 ± 5.89	(5)	0.667^b^

^a^ Independent two-sample *t*-test.

^b^ Mann–Whitney *U* test.

BMI, body mass index; DHSS, Dampness and heat in the spleen and stomach systems pattern; DLSS, Disharmony of liver and stomach systems pattern; EPS, epigastric pain syndrome; FR, food retention; FRED, food retention disorder pattern; NHT, *Naesohwajung-tang*; PDS, postprandial distress syndrome; SD, standard deviation; SDQS, spleen deficiency with *qi* stagnation pattern; SSDC, spleen and stomach deficiency with cold pattern; TACH, tangled cold and heat pattern; TDS, total dyspepsia symptom. Continuous values are presented as the mean ± SD. Statistically significant differences between the two groups were analyzed using the independent two-sample *t*-test or Mann–Whitney *U* test. *p < 0.05 and **p < 0.01 indicate statistically significant differences between the NHT and placebo groups.

### 3.3 Safety evaluation

All adverse events were documented in both groups during the study period. The safety analysis set included 116 participants from both groups. The total number of adverse events was 19 in the NHT group (12 patients) and 20 in the placebo group (13 patients). Information regarding the adverse events is shown in Table 6. One serious adverse event (SAE) occurred in each group, a breast mass in the NHT group and thyroid cancer in the placebo group, but both were judged to be unrelated to the study drugs owing to the onset of the disease. Analysis of the incidence of other drug-induced adverse events showed no significant difference between the study groups (*p* > 0.05), with all symptoms at a mild or moderate level of recovery without sequelae. In addition, no clinically significant abnormalities were observed in the vital signs, blood test results, or urinalysis results before and after drug administration.

**TABLE 6:** Adverse events in the NHT and placebo groups.

Items	NHT	Placebo	*p*-value
Number of subjects	58	58	
Yes, *n* (%)	12 (20.7)	13 (22.4)	0.821^a^
No, *n* (%)	46 (79.3)	45 (77.6)	
Number of events	19	20	
Types of adverse events (*n*)	Dyspepsia aggravated (2)	Dyspepsia aggravated (2)	
	Nausea (2)	Nausea (2)	
	Headache (2)	Headache (2)	
	Diarrhea (1)	Diarrhea (1)	
	Dizziness (1)	Constipation (1)	
	Heartburn (1)	Abdominal pain NOS (1)	
	Throat discomfort (1)	Periumbilical bruising (1)	
	Cervicalgia (1)	Low back pain (1)	
	Myalgia (1)	Coccyx pain (1)	
	Acute pharyngitis (1)	Common cold (5)	
	Arthritis (1)	Exposure to communicable disease (1)	
	Feelings of weakness (1)	Accident (1)	
	General physical condition decreased (1)	Thyroid cancer (1) (SAE)	
	Cystitis (1)		
	Traffic accident (1)		
	Breast mass (1) (SAE)		

^a^ Pearson’s chi-squared test.

NHT, *Naesohwajung-tang*; SAE, serious adverse event. *p* < 0.05 indicates statistically significant differences between the NHT and placebo groups.

## 4 Discussion

### 4.1 Summary of the evidence

Dyspepsia, characterized by pain or a heavy feeling in the stomach and postprandial discomfort, is a relatively common symptom. However, it is accompanied by a wide range of differential diagnoses and heterogeneous pathological mechanisms (Ford et al., 2020). As FD has therapeutic limitations as a multifactorial disease in which symptoms are inconsistent and do not individually correspond to the mechanism one by one (Miwa, 2012), various FD symptoms must be managed more effectively with multiple drugs for combination therapy than with a single drug (Wagner, 2006). An advantage of herbal medicines is that they can simultaneously target multiple pathophysiological mechanisms. Herbal medicines used to treat FD exert combined pharmacological effects related to motility and secretory activity in the GI tract (Gwee et al., 2021). According to the 2012 Asian guidelines (Miwa et al., 2012), herbal medicine has been suggested as a potential treatment option for FD. A recent network meta-analysis of 12 different herbal medicines in 15 RCTs showed that Xiao Yao Pill and Modified Ban Xia Xie Xin Decoction were significantly more effective than the placebo in improving dyspepsia-related symptoms (Ho et al., 2022).

NHT is a traditional herbal remedy frequently used in Korea for the treatment of FD. Numerous studies have explored the effects of botanical drugs in NHT on digestion (Supplementary Table S3). It is a combination of two herbal formulas, NSS and DHJ, which are prescribed for indigestion, abdominal distension, and loss of appetite, along with Atractylodis Rhizoma, Agastachis Herba, Aucklandiae Radix, Glycyrrhizae Radix et Rhizoma, and Zingiberis Rhizoma Recens (Yoon et al., 2001). Researchers have conducted several experiments using animal and cellular models to elucidate its therapeutic effects and mechanisms of action. NSS comprises 11 botanical drugs that promote gastric emptying through the cholinergic pathway in antral-dilated mice (Kim and Yoon, 2008). Anti-inflammatory effects have also been observed in experiments on rats with indomethacin-induced gastroinflammation (Park and Baik, 2013). DHJ, composed of seven botanical drugs, inhibits smooth muscle contractions induced by acetylcholine chloride and barium chloride in isolated ileum, colon, and fundus strips of rats. In addition, it has been shown to prevent pyloric ulcers induced by indomethacin and hydrochloride ethanol in rats (Yang et al., 1997). Furthermore, according to a study that analyzed the effect of NHT on gastric motility in rats, NHT significantly improved GMA and promoted gastric emptying, which were inhibited by N^G^-nitro-*L*-arginine methyl ester (Kim, 2007). These studies indicate that NHT, combined with the efficacy of the above two prescriptions, can treat FD by promoting GI motility to improve gastric dysmotility and adaptive relaxation disorders, and prevent inflammation against gastric mucosal injury.

To the best of our knowledge, this is the first randomized, double-blind, placebo-controlled clinical trial to show that NHT alleviates dyspeptic symptoms in patients with FD. In our study, significant differences were observed in TDS at week 4 and OTE at weeks 2, 4, and 8 in the NHT group compared with the placebo group. This means that the overall dyspepsia symptoms significantly improved after a 4 weeks administration of NHT, and the patient’s satisfaction with symptom relief was maintained even 4 weeks after the end of treatment. Moreover, compared to placebo, not only did the TDS score show a significant change at both 2 and 4 weeks, but also significant improvements were demonstrated in the symptoms of epigastric burning, postprandial fullness, early satiation, and FD-QoL scale after 4 weeks of NHT administration. The improvement degree of the DQ score in the NHT group was significantly greater than that in the placebo group after 4 weeks. Among the symptoms of FD, there was no significant difference in the degree of improvement of epigastric pain between the two groups. According to recent treatment guidelines for FD, prokinetics or PPIs are selectively administered as an initial choice for either PDS or EPS, depending on the main symptom (Vanheel and Tack, 2014). Similarly, it is necessary to consider different applications of herbal medicines for different patterns and symptoms in patients with FD.

In our study, we performed EGG tests on patients with FD corresponding to the PDS and overlap subtypes to analyze their GMA related to dysmotility. In addition to subjective symptoms, this objective evaluation provides a quantitative analysis of the clinical characteristics of patients with FD. The results showed that group administered NHT for 4 weeks had a less significant decrease in the percentage of normogastria after meals than the placebo group. There was a greater increase in the postprandial percentages of bradygastria, tachygastria, and arrhythmia in the placebo group than in the NHT group. However, the analysis of the results was based on only a few remaining clean data because a large number of subjects with missing values were excluded from the analysis. Bias in the results owing to missing information makes it difficult to generalize the outcomes. In addition, although a decreasing pattern in the percentage of normogastria was observed in the fed state compared to that in the fasted state in both groups, all the values satisfied the normal ranges of dominant frequency (2.0–4.0 cycles per minute) (Chen and McCallum, 1992), normal percentage of gastric slow wave (70%), and power ratio (>1) (Yin and Chen, 2013). For more consistent data collection in the future, it is necessary to completely test and retest measurements to minimize recording errors for a larger number of subjects and to determine significant values for each channel. Clinical considerations for further research regarding the duration of illness and the severity of the patient’s health status should be made for data analysis and interpretation.

As a result of the sub-analyses for the degree of improvement of the TDS score, NHT was more effective for FD patients who were female, under the age of 65 years, with a BMI of 22 or higher, overlap type, FR type, and DHSS. In TKM, pattern identification is the process of overall analysis of clinical data to determine the location, cause, and nature of a patient’s disease and to diagnose a pattern/syndrome (World Health Organization, 2007). It is used for diagnostic purposes and guides treatment principles based on a patient’s symptoms and signs (Berle et al., 2010). FR (Park et al., 2013) and DHSS types (Ha, 2020) commonly present feelings of fullness in the stomach and burning in the upper abdomen, corresponding to the overlap type of FD. Among the herbal prescriptions widely used for FD, for instance, previous studies have shown that Ban Xia Xie Xin Decoction for patients with FD of TACH (Zhao et al., 2013) and Xiao Yao San for SDQS (Qin et al., 2009) improved dyspeptic symptoms. This indicates that multiple prescriptions can be suggested depending on the type and pattern of the disease, even for similar symptoms. This differentiated therapeutic approach can help increase the effectiveness of herbal medicines in patients with FD who experience limitations with conventional treatments. In future studies, a detailed analysis will be required to evaluate the characteristics of patients with FD who show a positive response to the NHT.

In terms of safety, NHT had no noticeable adverse events compared with the placebo. The NHT and placebo groups had similar numbers and types of adverse events; their severity was mild and resolved without worsening. Although there was one SAE in each group, there was no definite connection to the study drug, thus confirming the safety of NHT.

### 4.2 Comparison with previous studies

Based on a systematic review of 26 RCTs aimed at determining the extent and underlying factors of the placebo response in FD, we found that the overall placebo response rate in pharmacological trials for FD was approximately 39.6% (Bosman et al., 2023). The placebo effect is thought to be influenced by psychological, neurobiological, and natural mechanisms (Jones and Holtmann, 2023). As lower baseline symptom scores are significantly related to higher placebo responses, proper entry criteria based on symptom severity should be established (Bosman et al., 2023). To address these limitations, we enrolled patients with FD with moderate or severe dyspeptic symptom scores (VAS ≥ 40). The SDS scale, which consists of four cardinal dyspeptic symptoms, was used as a secondary outcome measure. Additionally, we established a 4 week follow-up evaluation period after the administration of the investigational products to assess the sustained treatment effect in the absence of interventions.

Previous studies on the use of NHT for the treatment of FD have not compared its efficacy with that of a placebo in a clinical setting. Studies in animals, cells, and patients have shown that NHT can relieve dyspeptic symptoms and treat FD related to gastric dysmotility. This recent clinical trial confirmed that NHT administration could improve dyspepsia symptoms, quality of life, and GMA values; however, the reliability of the NHT findings in this study needs to be merged with further clinical and experimental evidence to verify other underlying mechanisms.

### 4.3 Limitations

This study has several limitations. First, the study duration was limited to 4 weeks, and there was a lack of long-term follow-up. According to the FD guidelines for conducting clinical trials, considering the prognosis of FD and the pharmacological functions of drugs, at least 4 weeks of treatment is recommended to assess short-term therapeutic effects (Lee et al., 2018). This 4 weeks treatment period is common in previous studies on FD using PPIs, prokinetics, and other herbal therapies (Jee et al., 2011; Xiao et al., 2012). However, as FD is prone to developing into a chronic disease in which symptoms change over time and recur periodically, further research is needed on the long-term safety and efficacy of NHT. Second, in addition to GMA measurement through EGG, other objective parameters related to GI motility, such as plasma levels of ghrelin and motilin, must also be analyzed. Furthermore, establishing a correlation between objective and other subjective evaluations in a sufficient number of participants with FD would enhance the credibility and clinical applicability of these findings. In the future, it will be important to elucidate the pathogenesis by which the active components of NHT relieve FD symptoms. Finally, this trial was conducted only in Korea, and further assessment of its external validity is required.

### 4.4 Strengths and future perspectives

Nevertheless, as the first RCT to evaluate the safety and therapeutic effects of NHT in patients with FD, the outcomes of this trial can reinforce the use of NHT for the treatment of FD. In this RCT, the results showed that after a 4 weeks treatment with NHT in patients with FD, symptom severity and quality of life significantly improved compared with placebo. There were no clinically significant differences in the incidence of adverse events between the two groups. This evidence can be used to develop clinical practice guidelines for the effective treatment and management of FD, which can help promote public health.

## 5 Conclusion

NHT is one of the most frequently prescribed herbal medicines to treat FD in the clinical settings of TKM. In this study, conducted with a rigorous RCT design, dyspeptic symptoms in patients with FD were alleviated after 4 weeks of NHT administration. In conclusion, our study showed that the herbal formula of NHT could be used as an effective and safe alternative therapy for FD. Further large-scale multinational clinical trials of NHT as an effective herbal medicine for FD or in combination with other therapies are needed.

## Figures and Tables

**Table:** 

**CONSORT**	Consolidated standards of reporting trials
**CRIS**	Clinical research information service
**DHJ**	*Daehwajung-eum*
**DHSS**	Dampness and heat in the spleen and stomach systems pattern
**DLSS**	Disharmony of liver and stomach systems pattern
**DP**	Dominant power
**DQ**	*Damum* questionnaire
**EGG**	Electrogastrography
**EPS**	Epigastric pain syndrome
**FAS**	Full analysis set
**FD**	Functional dyspepsia
**FD-QoL**	Functional dyspepsia-related quality of life
**FGID**	Functional gastrointestinal disorder
**FR**	Food retention
**FRED**	Food retention disorder pattern
**FRQ**	Food retention questionnaire
**GI**	Gastrointestinal
**GMA**	Gastric myoelectrical activity
**H**_**2**_ **RA**	H_2_ receptor antagonist
**KGMP**	Korean good manufacturing practice
**KHP**	Korean herbal pharmacopoeia
**KP**	Korean pharmacopoeia
**NHT**	*Naesohwajung-tang*
**NSS**	*Naeso-san*
**OTE**	Overall treatment effect
**PDS**	Postprandial distress syndrome
**PIFD**	Pattern identification of functional dyspepsia
**PPI**	Proton pump inhibitor
**RCT**	Randomized controlled trial
**SAE**	Serious adverse event
**SD**	Standard deviation
**SDQS**	Spleen deficiency with *qi* stagnation pattern
**SDS**	Single dyspepsia symptom
**SSDC**	Spleen and stomach deficiency with cold pattern
**TACH**	Tangled cold and heat pattern
**TDS**	Total dyspepsia symptom
**TKM**	Traditional Korean medicine
**VAS**	Visual analog scale

## Data Availability

The original contributions presented in the study are included in the article/Supplementary Material, further inquiries can be directed to the corresponding author.
