# Supplementary material for: Efficacy and safety of the herbal formula Naesohwajung-tang for functional dyspepsia: a randomized, double-blind, placebo-controlled, multi-center trial
Source: Front Pharmacol. 2023 May 12;14:1157535. doi: 10.3389/fphar.2023.1157535 (PMC10213234; doi:10.3389/fphar.2023.1157535)
Supplement: Supplementary file 1 [file Table1.docx]

**Supplementary Material**

Efficacy and safety of the herbal formula *Naesohwajung-tang* for functional dyspepsia: a randomized, double-blind, placebo-controlled, multi-center trial

Na-Yeon Ha, Seok-Jae Ko, Jae-Woo Park, Jinsung Kim*

* **Corresponding Author:** Jinsung Kim, KMD, PhD, oridoc@khu.ac.kr

Supplementary Table 1: Comparison of baseline EGG parameters between the NHT and placebo groups

| **Parameters** | **Ch** | **Mean ± SD (n)** | | | | ***^*^P* value** |
| --- | --- | --- | --- | --- | --- | --- |
|  |  | **NHT (19)** | ***^#^P* value** | **Placebo (18)** | ***^#^P* value** |  |
| Preprandial |  |  |  |  |  |  |
| DF (cpm) |  | 3.05 ± 0.17 |  | 2.92 ± 0.25 |  | 0.092^b^ |
| DP (dB) |  | 47.04 ± 5.31 |  | 46.68 ± 5.47 |  | 0.841^a^ |
| Percentage of normogastria | Ch1 | 89.30 ± 11.64 |  | 89.63 ± 9.14 |  | 0.924^a^ |
| (2.0–4.0 cpm) | Ch2 | 90.01 ± 11.28 |  | 90.19 ± 11.23 |  | 0.799^b^ |
|  | Ch3 | 90.17 ± 11.08 |  | 89.08 ± 13.32 |  | 0.869^b^ |
| Percentage of bradygastria | Ch1 | 1.93 ± 3.74 |  | 1.11 ± 2.80 |  | 0.620^b^ |
| (0.5–2.0 cpm) | Ch2 | 0.52 ± 1.24 |  | 0.55 ± 1.27 |  | 0.964^b^ |
|  | Ch3 | 0.70 ± 1.76 |  | 2.22 ± 3.96 |  | 0.327^b^ |
| Percentage of tachygastria | Ch1 | 1.76 ± 3.22 |  | 0.55 ± 1.27 |  | 0.499^b^ |
| (4.0–9.0 cpm) | Ch2 | 1.57 ± 2.32 |  | 1.11 ± 2.55 |  | 0.461^b^ |
|  | Ch3 | 1.58 ± 2.58 |  | 2.59 ± 4.05 |  | 0.620^b^ |
| Percentage of arrhythmia | Ch1 | 7.02 ± 7.20 |  | 8.69 ± 8.26 |  | 0.558^b^ |
|  | Ch2 | 7.90 ± 9.51 |  | 8.14 ± 10.17 |  | 0.988^b^ |
|  | Ch3 | 7.55 ± 9.74 |  | 6.11 ± 9.51 |  | 0.538^b^ |
| Postprandial |  |  |  |  |  |  |
| DF (cpm) |  | 2.94 ± 0.39 | 0.159^c^ | 3.03 ± 0.32 | 0.231^c^ | 0.443^a^ |
| DP (dB) |  | 47.12 ± 4.11 | 0.947^c^ | 47.68 ± 4.47 | 0.417^c^ | 0.693^a^ |
| Percentage of normogastria | Ch1 | 76.04 ± 9.41 | 0.001^c##^ | 83.71 ± 11.09 | 0.097^c^ | 0.029^a*^ |
| (2.0–4.0 cpm) | Ch2 | 76.59 ± 13.20 | 0.000^c###^ | 81.49 ± 12.86 | 0.024^c#^ | 0.261^a^ |
|  | Ch3 | 82.01 ± 13.14 | 0.069^c^ | 80.94 ± 12.74 | 0.030^c#^ | 0.641^b^ |
| Percentage of bradygastria | Ch1 | 3.84 ± 3.56 | 0.156^c^ | 2.41 ± 3.76 | 0.334^d^ | 0.142^b^ |
| (0.5–2.0 cpm) | Ch2 | 2.62 ± 2.38 | 0.010^d#^ | 3.69 ± 4.42 | 0.003^d##^ | 0.730^b^ |
|  | Ch3 | 3.14 ± 3.59 | 0.013^d#^ | 1.85 ± 3.08 | 0.672^d^ | 0.258^b^ |
| Percentage of tachygastria | Ch1 | 7.34 ± 5.29 | 0.002^c##^ | 3.14 ± 3.13 | 0.007^d##^ | 0.022^b*^ |
| (4.0–9.0 cpm) | Ch2 | 7.85 ± 6.50 | 0.001^c##^ | 3.89 ± 4.32 | 0.039^c#^ | 0.057^b^ |
|  | Ch3 | 6.10 ± 7.00 | 0.012^d#^ | 8.88 ± 8.86 | 0.001^d##^ | 0.284^b^ |
| Percentage of arrhythmia | Ch1 | 12.76 ± 7.29 | 0.005^c##^ | 10.73 ± 9.47 | 0.510^c^ | 0.271^b^ |
|  | Ch2 | 12.93 ± 9.29 | 0.012^c#^ | 10.91 ± 10.16 | 0.422^c^ | 0.499^b^ |
|  | Ch3 | 8.74 ± 8.55 | 0.708^c^ | 8.32 ± 6.41 | 0.350^c^ | 0.845^b^ |
| Power ratio | Ch1 | 1.04 ± 0.92 |  | 2.51 ± 6.63 |  | 0.799^b^ |
|  | Ch2 | 1.20 ± 1.03 |  | 1.02 ± 0.92 |  | 0.461^b^ |
|  | Ch3 | 3.77 ± 6.47 |  | 2.23 ± 2.39 |  | 0.775^b^ |

Ch, channel; CPM, cyclic per minute; DF, dominant frequency; DP, dominant power; EGG, electrogastrography; NHT, *Naesohwajung-tang*; SD, standard deviation.

Statistically significant differences between the two groups were analyzed using the independent two-sample *t*-test or Mann–Whitney *U* test.

Statistically significant differences between the pre- and postprandial values were analyzed using the paired sample *t*-test or Wilcoxon signed-rank test.

Continuous values are presented as the mean ± SD.

^*^*P* < 0.05 indicates statistically significant differences between the NHT and placebo groups.

^#^*P* < 0.05, ^##^*P* < 0.01, and ^###^*P* < 0.001 indicate statistically significant differences compared with the preprandial values.

^a^ Independent two-sample *t*-test.

^b^ Mann–Whitney *U* test.

^c^ Paired sample *t*-test.

^d^ Wilcoxon signed-rank test.

Supplementary Table 2: Comparison of EGG parameters after treatment (week 4) between the NHT and placebo groups

| **Parameters** | **Ch** | **Mean ± SD (n)** | | | | ***^*^P* value** |
| --- | --- | --- | --- | --- | --- | --- |
|  |  | **NHT (24)** | ***^#^P* value** | **Placebo (26)** | ***^#^P* value** |  |
| Preprandial |  |  |  |  |  |  |
| DF (cpm) |  | 3.05 ± 0.16 |  | 3.00 ± 0.20 |  | 0.405^b^ |
| DP (dB) |  | 46.24 ± 5.46 |  | 45.83 ± 5.74 |  | 0.797^a^ |
| Percentage of normogastria | Ch1 | 83.91 ± 14.68 |  | 89.89 ± 11.26 |  | 0.161^b^ |
| (2.0–4.0 cpm) | Ch2 | 82.80 ± 13.07 |  | 90.92 ± 7.81 |  | 0.012^a*^ |
|  | Ch3 | 88.08 ± 13.66 |  | 86.60 ± 13.46 |  | 0.666^b^ |
| Percentage of bradygastria | Ch1 | 1.52 ± 2.78 |  | 1.02 ± 1.82 |  | 0.777^b^ |
| (0.5–2.0 cpm) | Ch2 | 1.65 ± 3.10 |  | 0.76 ± 1.68 |  | 0.267^b^ |
|  | Ch3 | 1.38 ± 2.38 |  | 1.65 ± 3.13 |  | 0.857^b^ |
| Percentage of tachygastria | Ch1 | 2.77 ± 4.58 |  | 1.79 ± 2.54 |  | 0.586^b^ |
| (4.0–9.0 cpm) | Ch2 | 5.56 ± 9.20 |  | 1.66 ± 3.79 |  | 0.040^b*^ |
|  | Ch3 | 2.08 ± 3.38^c^ |  | 2.42 ± 3.34 |  | 0.663^b^ |
| Percentage of arrhythmia | Ch1 | 11.79 ± 11.64 |  | 7.29 ± 8.70 |  | 0.169^b^ |
|  | Ch2 | 9.95 ± 8.40 |  | 6.66 ± 6.80 |  | 0.154^b^ |
|  | Ch3 | 8.45 ± 11.31 |  | 9.30 ± 9.61 |  | 0.378^b^ |
| Postprandial |  |  |  |  |  |  |
| DF (cpm) |  | 3.01 ± 0.39 | 0.669^c^ | 2.92 ± 0.34 | 0.181^c^ | 0.347^a^ |
| DP (dB) |  | 46.03 ± 5.45 | 0.871^c^ | 46.70 ± 4.71 | 0.476^c^ | 0.647^a^ |
| Percentage of normogastria | Ch1 | 80.39 ± 12.88 | 0.395^c^ | 77.31 ± 12.00 | 0.000^c###^ | 0.275^b^ |
| (2.0–4.0 cpm) | Ch2 | 78.27 ± 13.19 | 0.246^c^ | 78.07 ± 14.09 | 0.000^c###^ | 0.959^a^ |
|  | Ch3 | 78.03 ± 14.05 | 0.002 ^c##^ | 75.64 ± 13.52 | 0.001^c##^ | 0.543^a^ |
| Percentage of bradygastria | Ch1 | 4.15 ± 4.42 | 0.027^c#^ | 3.84 ± 5.56 | 0.014^d#^ | 0.539^b^ |
| (0.5–2.0 cpm) | Ch2 | 5.24 ± 5.34 | 0.025^c#^ | 5.25 ± 5.67 | 0.000^d###^ | 0.976^b^ |
|  | Ch3 | 4.15 ± 3.82 | 0.003^d##^ | 6.02 ± 5.66 | 0.003^c##^ | 0.294^b^ |
| Percentage of tachygastria | Ch1 | 4.68 ± 5.66 | 0.273^c^ | 6.02 ± 5.89 | 0.003^c##^ | 0.302^b^ |
| (4.0–9.0 cpm) | Ch2 | 4.14 ± 5.02 | 0.810^d^ | 5.52 ± 7.67 | 0.011^d#^ | 0.519^b^ |
|  | Ch3 | 6.37 ± 6.20 | 0.005^c##^ | 5.25 ± 6.20 | 0.092^d^ | 0.618^b^ |
| Percentage of arrhythmia | Ch1 | 10.76 ± 8.97 | 0.706^c^ | 12.83 ± 7.09 | 0.006^c##^ | 0.244^b^ |
|  | Ch2 | 12.32 ± 9.23 | 0.308^c^ | 11.15 ± 9.66 | 0.065^c^ | 0.531^b^ |
|  | Ch3 | 11.45 ± 9.75 | 0.124^c^ | 13.07 ± 9.48 | 0.077^c^ | 0.519^b^ |
| Power ratio | Ch1 | 2.67 ± 4.49 |  | 1.79 ± 2.67 |  | 0.697^b^ |
|  | Ch2 | 2.31 ± 3.00 |  | 1.84 ± 2.31 |  | 0.409^b^ |
|  | Ch3 | 2.23 ± 2.05 |  | 2.20 ± 2.08 |  | 0.600^b^ |

Ch, channel; CPM, cyclic per minute; DF, dominant frequency; DP, dominant power; EGG, electrogastrography; NHT, *Naesohwajung-tang*; SD, standard deviation.

Statistically significant differences between the two groups were analyzed using the independent two-sample *t*-test or Mann–Whitney *U* test.

Statistically significant differences between the pre- and postprandial values were analyzed using the paired sample *t*-test or Wilcoxon signed-rank test.

Continuous values are presented as the mean ± SD.

^*^*P* < 0.05 indicates statistically significant differences between the NHT and placebo groups.

^#^*P* < 0.05, ^##^*P* < 0.01, and ^###^*P* < 0.001 indicate statistically significant differences compared with the preprandial values.

^a^ Independent two-sample *t*-test.

^b^ Mann–Whitney *U* test.

^c^ Paired sample *t*-test.

^d^ Wilcoxon signed-rank test.

Supplementary Table 3: Pharmacological actions of single components in NHT

| **No.** | **Scientific name**  **(family)** | **Chinese name** | **Common name** | **Latin name** | **Parts and form used** | **Dosage per serving (g)** | **Impact on digestive system** |
| --- | --- | --- | --- | --- | --- | --- | --- |
| 1 | *Crataegus pinnatifida* Bunge  (family *Rosaceae*) | Shanzha | Hawthorn fruit | *Crataegi Fructus* | dried ripe fruit | 2.50 | - Increase or decrease in contractility of murine gastric and intestinal smooth muscle (Huang et al., 2009; Wen et al, 2010)  - Promotion of secretion and activity of digestive enzymes in the stomach (Shen et al., 2000) |
| 2 | *Hordeum vulgare* L.  (family *Poaceae*) | Chao-Maiya | Barley sprouts | *Hordei Fructus Germinatus* | (stir-baked) dried germinated ripe fruit | 2.50 | - Regulation of brain-gut peptides and gut microbiota in mice (Wu et al., 2020) |
| 3 | *Magnolia officinalis* Rehder & E.H.Wilson  (family *Magnoliaceae*) | Houpo | Magnolia bark | *Magnoliae Cortex* | dried stem bark, root bark, or branch bark | 1.88 | - Promotion of gastrointestinal transit function in mice (Kim et al., 2017; Tian et al., 2015) |
| 4 | *Citrus reticulata* Blanco  (family *Rutaceae*) | Chenpi | Citrus unshiu peel | *Citri Unshius Pericarpium* | dried ripe pericarp | 1.88 | - Improving gastrointestinal motility in rats (Lyu and Lee, 2013) |
| 5 | *Alisma plantago-aquatica* subsp. *orientale* (Sam.) Sam.  (family *Alismataceae*) | Zexie | Alisma rhizome | *Alismatis Rhizoma* | dried tuber | 1.88 | - Improving the balance of gut microbes and intestinal barrier function in mice (Zhu et al., 2021) |
| 6 | *Atractylodes lancea* (Thunb.) DC.  (family *Compositae*) | Cangzhu | Atractylodes rhizome | *Atractylodis Rhizoma* | dried rhizome | 1.25 | - Anti-gastric ulcer effect in rats (Yu et al., 2015)  - Improving delayed gastric emptying in mice (Kimura and Sumiyoshi, 2012; Nakai et al., 2003) |
| 7 | *Cyperus rotundus* L.  (family *Cyperaceae*) | Xiangfu | Cyperus rhizome | *Cyperi Rhizoma* | dried rhizome | 1.25 | - Antidepressant effect in rats (Lu et al., 2022) |
| 8 | *Citrus trifoliata* L.  (family *Rutaceae*) | Zhishi | Poncirus immature fruit | *Ponciri Fructus Immaturus* | dried unripe fruit | 1.25 | - Relaxation of gastric fundus smooth muscle in rats (Kim, 2013) |
| 9 | *Pinellia ternata* (Thunb.) Makino  (family *Araceae*) | Banxia | Pinellia tuber | *Pinelliae Tuber* | dried tuber | 1.25 | - Antiemetic effect of leopard and ranid frogs (Marki et al., 1987)  - Enhancement of efferent activity of the gastric vagus nerve in rats (Niijima et al., 1993)  - Sedative effect of mice (Wu et al., 2011) |
| 10 | *Poria cocos* (Schw.) Wolf.  (family *Polyporaceae*) | Fuling | Poria | *Poria Sclerotium* | dried sclerotium | 1.25 | - Antiemetic effect (Tai et al., 1995)  - Improving intestinal barrier function in vitro (Xu et al., 2019a)  - Modulation of gut microbiota and mitigation of intestinal damage in mice (Zou et al., 2021) |
| 11 | - | Chao-Shenqu | Fermented mixture | *Massa Medicata Fermentata* | (stir-baked) fermented mixture of botanical drugs (Zhang et al., 2022) | 1.25 | - Amylase activity in vitro (Xu et al., 2019b)  - Improving the relaxation of the proximal stomach and modulating the disturbance of the gut microbiota in mice (Zhang et al., 2020) |
| 12 | *Wurfbainia villosa* (Lour.) Škorničk. & A.D.Poulsen  (family *Zingiberaceae*) | Sharen | Amomum fruit | *Amomi Fructus* | dried ripe fruit | 1.25 | - Promotion of gastrointestinal motility in dogs (Chen et al., 2016)  - Enhancing the intestinal mucosal barrier function in mice (Zhang et al., 2017) |
| 13 | *Sparganium stoloniferum* (Buch.-Ham. ex Graebn.) Buch.-Ham. ex Juz.  (family *Typhaceae*) | Sanleng | Sparganium rhizome | *Sparganii Rhizoma* | dried rhizome | 1.25 | - Analgesic effect in mice (Lu et al., 1997) |
| 14 | *Curcuma phaeocaulis* Valeton  (family *Zingiberaceae*) | Ezhu | Zedoary | *Curcumae Rhizoma* | dried rhizome | 1.25 | - Anticancer activity in vitro (Liu et al., 2011; Shi et al., 2013) |
| 15 | *Zingiber officinale* Roscoe  (family *Zingiberaceae*) | Ganjiang | Dried ginger | *Zingiberis Rhizoma* | dried rhizome | 1.25 | - Antiemetic activity in cisplatin-induced vomiting dogs (Sharma et al., 1997)  - Anti-gastric ulcer effect in rats (Yamahara et al., 1988)  - Stimulation of bile secretion in rats (Yamahara et al., 1985) |
| 16 | *Zingiber officinale* Roscoe  (family *Zingiberaceae*) | Shengjiang | Fresh ginger | *Zingiberis Rhizoma Recens* | fresh rhizome | 1.25 | - Antiemetic effect (Li et al., 2021)  - Inhibition of ileal contraction in guinea pigs in vitro (Du et al., 2016) |
| 17 | *Agastache rugosa* (Fisch. & C.A.Mey.) Kuntze  (family *Lamiaceae*) | Huoxiang | Agastache | *Agastachis Herba* | dried aerial part | 1.00 | - Promotion of isometric contraction of ileal smooth muscle and anti-gastric ulcer activity in rats (Jo et al., 1996) |
| 18 | *Aucklandia lappa* DC.  (family *Compositae*) | Muxiang | Aucklandia root | *Aucklandiae Radix* | dried root | 0.67 | - Improvement of gastric ulcer in mice (Han et al., 2005)  - Inhibition of the gastric emptying and intestinal transit in neostigmine-induced mice, and inhibit of rat jejunum contraction in vitro (Guo et al., 2014) |
| 19 | *Glycyrrhiza uralensis* Fisch.  (family *Leguminosae*) | Gancao | Licorice root | *Glycyrrhizae Radix et Rhizoma* | dried root and rhizome | 0.67 | - Anti-gastric ulcer effect in mice (Liu et al., 2021)  - Promotion of secretin release (Watanabe et al., 1986) |

NHT, *Naesohwajung-tang*.

**References**

Chen, Q., Pang, F., Cao, L., Jiang, Z., Zhou, L., and Chen, Z. (2016). Effect of Chinese herbs on gastrointestinal motility of chronic experimental beagle model. *J. Guangzhou Univ. Trad. Chin. Med.* 33 (5)**,** 674-678. doi:10.13359/j.cnki.gzxbtcm.2016.05.014

Du, J., Zhang, Q., Li, G., and Nie, K. (2016). Effects of rhizoma zingiberis recens on 5-HT3 receptor mediated contractile activity of isolated Guinea-pig ileum. *Trad. Chin. Drug Res. Clin. Pharmacol.* 27 (5)**,** 632-636. doi:10.19378/j.issn.1003-9783.2016.05.008

Guo, H., Zhang, J., Gao, W., Qu, Z., and Liu, C. (2014). Gastrointestinal effect of methanol extract of Radix Aucklandiae and selected active substances on the transit activity of rat isolated intestinal strips. *Pharm. Biol.* 52 (9)**,** 1141-1149. doi:10.3109/13880209.2013.879601

Han, J., Lin, H., Zhong, Z., and Rong, X. (2005). Study of the effect of exceed critical extracts from Radix Aucklandiae on experimental gastric ulcer model. *J. Chin. Med. Mater.* 11**,** 1017-1019. doi:10.13863/j.issn1001-4454.2005.11.021

Huang, S., Lin, Y., Diao, Y., Liu, Z., Zhen, Y., and Zhang, H. (2009). Effects of charred fructus Crataegi alcohol extract on contractility of isolated rat gastric and intestine muscle strips. *Prog. Mod. Biomed.* 4**,** 612-614. https://www.cnki.com.cn/Article/CJFDTOTAL-SWCX200904005.htm

Jo, S. G., Park, H. R., and Kim, C. J. (1996). Spasmolytic and Anti-peptic Ulcer Activities of Crude Drugs Acting on Gastrointestinal Tract in Rats. *J. Pharm. Soc. Kor.* 40 (5)**,** 591-598. https://koreascience.kr/article/JAKO199615875838894.page

Yamahara, J., Miki, K., Chisaka, T., Sawada, T., Fujimura, H., Tomimatsu, T., et al. (1985). Cholagogic effect of ginger and its active constituents. *J. Ethnopharmacol.* 13 (2)**,** 217-225. doi:10.1016/0378-8741(85)90009-1

Yamahara, J., Mochizuki, M., Rong, H. Q., Matsuda, H., and Fujimura, H. (1988). The anti-ulcer effect in rats of ginger constituents. *J. Ethnopharmacol.* 23 (2-3)**,** 299-304. doi:10.1016/0378-8741(88)90009-8

Kim, H. J., Han, T., Kim, Y. T., So, I., and Kim, B. J. (2017). Magnolia officinalis bark extract induces depolarization of pacemaker potentials through M2 and M3 muscarinic receptors in cultured murine small intestine interstitial cells of cajal. *Cell. Physiol. Biochem.* 43 (5)**,** 1790-1802. doi:10.1159/000484065

Kim, T. W. (2013). Effects of Ponciri Fructus and Aurantii Fructus Immaturus on the Gastric Fundus Motility. *J. Vet. Clin.* 30 (1)**,** 27-31. https://koreascience.kr/article/JAKO201310554376564.page

Kimura, Y. and Sumiyoshi, M. (2012). Effects of an Atractylodes lancea rhizome extract and a volatile component β-eudesmol on gastrointestinal motility in mice. *J. Ethnopharmacol.* 141 (1)**,** 530-536. doi:10.1016/j.jep.2012.02.031

Li, X., Ao, M., Zhang, C., Fan, S., Chen, Z., and Yu, L. (2021). Zingiberis Rhizoma Recens: a review of its traditional uses, phytochemistry, pharmacology, and toxicology. *Evid. Based Complement. Alternat. Med.* 2021, 1-20. doi:10.1155/2021/6668990

Liu, J., Zhang, Y., Qu, J., Xu, L., Hou, K., Zhang, J., et al. (2011). β-Elemene-induced autophagy protects human gastric cancer cells from undergoing apoptosis. *BMC cancer* 11**,** 1-10. doi:10.1186/1471-2407-11-183

Liu, Y., Zhang, M., Cheng, J., Zhang, Y., Kong, H., Zhao, Y., et al. (2021). Novel Carbon Dots Derived from Glycyrrhizae Radix et Rhizoma and Their Anti-Gastric Ulcer Effect. *Molecules* 26 (6)**,** 1512. doi:10.3390/molecules26061512

Lu, J., Li, W., Gao, T., Wang, S., Fu, C., and Wang, S. (2022). The association study of chemical compositions and their pharmacological effects of Cyperi Rhizoma (Xiangfu), a potential traditional Chinese medicine for treating depression. *J. Ethnopharmacol.* 287**,** 114962. doi:10.1016/j.jep.2021.114962

Lu, T., Mao, C., and Qiu, L. (1997). The research of analgestic action of different processed products of Sparganium stoloniferum. *J. Chin. Med. Mater.* 20 (3)**,** 135-137. doi:10.13863/j.issn1001-4454.1997.03.010

Lyu, J. H. and Lee, H. T. (2013). Effects of dried Citrus unshiu peels on gastrointestinal motility in rodents. *Arch. Pharm. Res.* 36 (5)**,** 641-648. doi: 0.1007/s12272-013-0080-z

Marki, T., Takahashi, K., and Shibata, S. (1987). An anti-emetic principle of Pinellia ternata tuber. *Planta Med.* 53 (5)**,** 410-414. doi:10.1055/s-2006-962759

Nakai, Y., Kido, T., Hashimoto, K., Kase, Y., Sakakibara, I., Higuchi, M., et al. (2003). Effect of the rhizomes of Atractylodes lancea and its constituents on the delay of gastric emptying. *J. Ethnopharmacol.* 84 (1)**,** 51-55. doi:10.1016/s0378-8741(02)00260-x

Niijima, A., Okui, Y., Kubo, M., Higuchi, M., Taguchi, H., Mitsuhashi, H., et al. (1993). Effect of Pinellia ternata tuber on the efferent activity of the gastric vagus nerve in the rat. *Brain Res. Bull.* 32 (2)**,** 103-106. doi:10.1016/0361-9230(93)90063-h

Sharma, S., Kochupillai, V., Gupta, S., Seth, S., and Gupta, Y. (1997). Antiemetic efficacy of ginger (Zingiber officinale) against cisplatin-induced emesis in dogs. *J. Ethnopharmacol.* 57 (2)**,** 93-96. doi:10.1016/s0378-8741(97)00054-8

Shen, Y. J., Ma, S. P., and Deng, W. L. (2000). Pharmacology of Chinese materia medica. *Beijing: People’s Medical Publishing House***,** 386-393.

Shi, H., Tan, B., Ji, G., Lu, L., Cao, A., Shi, S., et al. (2013). Zedoary oil (Ezhu You) inhibits proliferation of AGS cells. *Chin. Med.* 8 (13), 1-11. doi:10.1186/1749-8546-8-13

Tai, T., Akita, Y., Kinoshita, K., Koyama, K., Takahashi, K., and Watanabe, K. (1995). Anti-emetic principles of Poria cocos. *Planta Med.* 61 (6)**,** 527-530. doi:10.1055/s-2006-959363

Tian, H., Huang, D., Li, T., Huang, L., Zheng, X., Tang, D., et al. (2015). The protective effects of total phenols in magnolia officinalix rehd. et wils on gastrointestinal tract dysmotility is mainly based on its influence on interstitial cells of cajal. *Int. J. Clin. Exp. Med.* 8 (11)**,** 20279-86. https://www.ncbi.nlm.nih.gov/pmc/articles/PMC4723786

Watanabe, S. I., Chey, W. Y., Lee, K. Y., and Chang, T. M. (1986). Release of secretin by licorice extract in dogs. *Pancreas* 1 (5)**,** 449-454. doi:10.1097/00006676-198609000-00010

Wen, X., Deng, S., Lin, Y., Diao, Y., Huang, S., and Zhang, H. (2010). Effects of folium crataegi water extract on the contractility of isolated gastric and intestinal muscle strips in rats. *China Pharmacy*. 21 (11), 978-980. https://www.cnki.com.cn/Article/CJFDTOTAL-ZGYA201011010.htm

Wu, L., Lai, Y., Wang, Y., Tan, L., Wen, L., and Yang, H. (2020). Maillard reaction products of stir fried hordei fructus germinatus are important for its efficacy in treating functional dyspepsia. *J. Med. Food.* 23 (4)**,** 420-431. doi:10.1089/jmf.2019.4430

Wu, X. Y., Zhao, J. L., Zhang, M., Li, F., Zhao, T., and Yang, L. Q. (2011). Sedative, hypnotic and anticonvulsant activities of the ethanol fraction from Rhizoma Pinelliae Praeparatum. *J. Ethnopharmacol.* 135 (2)**,** 325-329. doi:10.1016/j.jep.2011.03.016

Xu, H., Wang, Y., Jurutka, P.W., Wu, S., Chen, Y., Cao, C., et al. (2019a). 16α-Hydroxytrametenolic acid from Poria cocos improves intestinal barrier function through the glucocorticoid receptor-mediated PI3K/Akt/NF-κB pathway. *J. Agric. Food Chem.* 67 (39)**,** 10871-10879. doi:10.1021/acs.jafc.9b04613

Xu, M. S., Fu, Q., and Baxter, A. (2019b). The components and amylase activity of Massa Medicata Fermentata during the process of fermentation. *Trends Food Sci. Technol.* 91**,** 653-661. doi:10.1016/j.tifs.2019.07.027

Yu, Y., Jia, T. Z., Cai, Q., Jiang, N., Ma, M. Y., Min, D. Y., et al. (2015). Comparison of the anti-ulcer activity between the crude and bran-processed Atractylodes lancea in the rat model of gastric ulcer induced by acetic acid. *J. Ethnopharmacol.* 160**,** 211-218. doi:10.1016/j.jep.2014.10.066

Zhang, H., Gao, S., Zhang, X., Meng, N., Chai, X., and Wang, Y. (2022). Fermentation characteristics and the dynamic trend of chemical components during fermentation of Massa Medicata Fermentata. *Arab. J. Chem.* 15 (1)**,** 103472. doi:10.1016/j.arabjc.2021.103472

Zhang, T., Lu, S. H., Bi, Q., Liang, L., Wang, Y. F., Yang, X. X., et al. (2017). Volatile oil from Amomi Fructus attenuates 5-fluorouracil-induced intestinal mucositis. *Front. Pharmacol.* 8**,** 786. doi:10.3389/fphar.2017.00786

Zhang, X., Zhang, H., Huang, Q., Sun, J., Yao, R., and Wang, J. (2020). Effect of Massa Medicata Fermentata on the gut microbiota of dyspepsia mice based on 16S rRNA technique. *Evid. Based. Complement. Alternat. Med.* 2020, 7643528. doi:10.1155/2020/7643528

Zhu, H. C., Jia, X. K., Fan, Y., Xu, S. H., Li, X. Y., Huang, M. Q., et al. (2021). Alisol B 23-Acetate Ameliorates Azoxymethane/Dextran Sodium Sulfate-Induced Male Murine Colitis-Associated Colorectal Cancer via Modulating the Composition of Gut Microbiota and Improving Intestinal Barrier. *Front. Cell. Infect. Microbiol.* 11, 640225. doi:10.3389/fcimb.2021.640225

Zou, Y. T., Zhou, J., Wu, C. Y., Zhang, W., Shen, H., Xu, J. D., et al. (2021). Protective effects of Poria cocos and its components against cisplatin-induced intestinal injury. *J. Ethnopharmacol.* 269**,** 113722. doi:10.1016/j.jep.2020.113722
